# Effectiveness of fall prevention interventions in residential aged care and community settings: an umbrella review

**DOI:** 10.1186/s12877-023-04624-4

**Published:** 2024-01-19

**Authors:** Isabelle Meulenbroeks, Crisostomo Mercado, Peter Gates, Amy Nguyen, Karla Seaman, Nasir Wabe, Sandun M Silva, Wu Yi Zheng, Deborah Debono, Johanna Westbrook

**Affiliations:** 1 Australian Institute of Health Innovation, Macquarie University, Level 6, 75 Talavera Rd North Ryde, Sydney, NSW 2113 Australia; 2 National Drug & Alcohol Research Centre, University of New South Wales, Sydney, NSW Australia; 3 St Vincent’s Clinical School, UNSW Medicine, UNSW Sydney, Sydney, NSW Australia; 4 Black Dog Institute, University of New South Wales, Sydney, NSW Australia; 5 School of Public Health, University of Technology Sydney, Sydney, NSW Australia

**Keywords:** Falls, Aged care, Community, Vitamin D, Multifactorial, Exercise, Older adults

## Abstract

**Introduction:**

Preventing falls is a priority for aged care providers. Research to date has focused on fall prevention strategies in single settings (e.g., residential aged care (RAC) or community settings). However, some aged care providers deliver care, including fall prevention interventions, across RAC and community settings. We conducted an umbrella review to identify what type of fall prevention interventions had the greatest impact on falls outcomes in RAC and community settings.

**Methods:**

Five databases were searched for systematic reviews of falls prevention randomised control trials in older adults living in the community or RAC. Data extracted included systematic review methods, population characteristics, intervention characteristics, setting details (RAC or community), and fall-related outcomes (falls, people who have had a fall, fall-related hospitalisations, and fall-related fractures). Review quality was appraised using the Assessment of Multiple Systematic Reviews-2 tool.

**Results:**

One-hundred and six systematic reviews were included; 63 and 19 of these stratified results by community and RAC settings respectively, the remainder looked at both settings. The most common intervention types discussed in reviews included ‘exercise’ (61%, *n* = 65), ‘multifactorial’ (two or more intervention types delivered together) (26%, *n* = 28), and ‘vitamin D’ (18%, *n* = 19). In RAC and community settings, ‘exercise’ interventions demonstrated the most consistent reduction in falls and people who have had a fall compared to other intervention types. ‘Multifactorial’ interventions were also beneficial in both settings however demonstrated more consistent reduction in falls and people who fall in RAC settings compared to community settings. ‘Vitamin D’ interventions may be beneficial in community-dwelling populations but not in RAC settings. It was not possible to stratify fall-related hospitalisation and fall-related fracture outcomes by setting due to limited number of RAC-specific reviews (*n* = 3 and 0 respectively).

**Conclusion:**

‘Exercise’ interventions may be the most appropriate falls prevention intervention for older adults in RAC and community settings as it is beneficial for multiple fall-related outcomes (falls, fall-related fractures, and people who have had a fall). Augmenting ‘exercise’ interventions to become ‘multifactorial’ interventions may also improve the incidence of falls in both settings.

**Supplementary Information:**

The online version contains supplementary material available at 10.1186/s12877-023-04624-4.

## Introduction

Falls—where a person inadvertently comes to rest on a lower level or the ground—are common and account for significant morbidity and mortality, placing substantial demands on the public health care system [1, 2]. Every year in Australia, 224,000 people are hospitalised due to falls and fall-related injuries [3]. The risk of falling increases with certain modifiable and non-modifiable factors, such as cognitive impairment, neurological conditions, and low physical activity [4]. Overall, older adults (≥60 years old) are most at risk of falling, with one in three community-dwelling older adults falling per year and institutionalised older adults fall approximately twice per year [2, 5, 6].

Falls have numerous consequences including fractures [2], fear-related avoidance of activities, poor quality of life [7], and in some cases death [8]. For community-dwelling older adults, falls are also a strong predictor of entry into residential aged care (RAC) (also known as nursing homes) [9]. In Australia, all falls result in an estimated 5300 deaths and cost approximately AU$8.9 billion per year [10]. As the number of falls increases over time [3, 11], preventing falls, should be a national priority.

Fall prevention interventions aim to reduce the risk and incidence of falling. Fall prevention interventions which have been found to significantly reduce the risk and rate of falling for older adults include exercise such as Tai Chi, strength, and balance programs, vitamin D prescription, and environmental modifications such as handrail installation and trip hazard removal [12–14]. However, it is important to recognise that falls in the older adult population are a multifaceted problem, multiple factors such as poor balance and function, cognitive decline, and high use of medications contribute to high falls risk. To address the multiple underlying factors, many fall prevention guidelines also recommend the use of multifactorial interventions which combine two or more intervention types [1, 6, 15–17]. Previous fall prevention intervention reviews have focused on a specific setting (e.g., community or RAC facilities) as this is how specific interventional studies are traditionally conducted [13, 18].

Providing recommendations for effective fall prevention interventions based on setting is important for aged care providers who provide both RAC and community aged care services. While there is an extensive number of reviews on fall prevention interventions, many are setting specific, and they do not synthesise or compare what type of interventions are most effective at improving fall-related outcomes in each setting. Important differences likely exist in fall prevention interventions in RAC and community setting due to environmental factors (e.g., staffing, and physical layout) and client factors (e.g., RAC residents fall more often than community-dwelling older adults). A direct comparison between fall prevention interventions in RAC and community settings may help the providers to target broad fall prevention programs to each setting. In this umbrella review, we aimed to summarise the highest quality evidence, systematic reviews of randomised control trials (RCTs), to identify which fall prevention interventions are the most effective for improving falls outcomes in community and RAC settings respectively.

## Methods

### Protocol

This umbrella review followed a protocol registered with PROSPERO (CRD42022306518). The study design was informed by the Joanna Briggs Institute Manual for Evidence Synthesis [19].

### Data source and search strategy

Five databases, Medline, EMBASE, Scopus, CENTRAL, and CINAHL, were systematically searched in November 2021 using the key terms “fall”, “elderly”, and “systematic review”. The search was updated in June 2023. The search strategy was informed by previous reviews [18] and our own preliminary searches. Search strategies are available in Appendix 1. All searches were translated to each database and restricted to articles published since 2000 and the English language. Additional manual searching was conducted by screening the reference lists of articles that underwent full-text screening (citation searching).

### Inclusion criteria

Systematic reviews of RCTs of fall prevention interventions in community and RAC settings were identified in this umbrella review. In this review, reviews were considered systematic if authors called the review a ‘systematic review’. Systematic reviews were included if the mean age of the population was ≥60 years old, they included RCT interventions which aimed to reduce falls and reported on fall outcomes (rate/number of falls, people who have had fall, falls requiring hospitalisation, and fall-related fractures) as a primary outcome.

Articles were excluded if they were not systematic reviews, such as a scoping review or narrative reviews, conference abstracts, studied populations with acute medical conditions or within acute or subacute settings, published before 2000, or were written in a language other than English. Articles were also excluded if fall-related outcomes were not the primary outcome. The inclusion was limited to 2000 onwards for quality purposes as reporting standards for meta-analyses and systematic reviews were first constructed in 1999 [20].

### Screening

Article screening was conducted in two steps, title/abstract and full-text screening, and used Rayyan [21], a web-based artificial intelligence platform which facilitates manual searching by highlighting key inclusion/exclusion criteria in the text. Two reviewers (IM, AN) independently screened 10% of articles at each stage to reach consistency in the application of the inclusion and exclusion criteria. Inter-rater reliability between reviewers was high (title/abstract screening: k = 0.96, full text screening: k = 0.79). Conflicts in screening were discussed in regular team meetings. The remaining 90% of articles were screened by one reviewer (IM).

### Data extraction

Data were extracted by five reviewers (IM, AN, PG, CM, JS) using a purpose designed Excel spreadsheet. The data extraction sheet was piloted by all reviewers on a sample of included articles to inform extraction sheet design and ensure consistency in data extraction. Data extracted included review methods (e.g., number of databases searched, quality appraisal used, and review steps conducted in duplicate), participant information (e.g., setting, number), control group, and results (e.g., meta-analysis results or summary of narrative results, adverse events, range of length and intensity of intervention, and subgroup analyses). All data collected and entered arose from the systematic reviews, and not the RCTs discussed within included systematic reviews.

Data collected on interventions, outcomes, and comparator groups were categorised to aid data synthesis. Categories are defined in Table 1. The data extraction sheet and allocation to categories were checked and cleaned by two researchers (IM, PG). Outcome data collected were also categorised as significant, non-significant, and no difference (definitions in Table 1). Multiple outcomes were extracted from reviews when they studied more than one outcome category, intervention type, and/or population group.

**Table 1:** Definitions for data extraction

Category	Definition
Outcome terms	
*Falls*	Any measure (e.g., count, rate) of every fall within the population.
*People who have had one or more falls*	Any measure of people who have fallen once or more times including count and rate. In the literature this outcome is commonly termed fallers.
*Falls requiring hospitalisation*	Any measure of falls which required a hospital stay (emergency department visit alone was excluded) including count and rate.
*Fall-related fractures*	Any measure of falls resulting in fracture including count and rate. Fractures which are not fall-related were excluded from this measure.
Comparator group terms	
*Active*	Where control groups received an intervention of lesser intensity than the intervention group e.g., single home visit compared to multiple visits or assessment only compared to multi-visit exercise intervention.
*Passive*	Where the control group receives usual care which often involves some care such as routine interventions in a residential aged care home or fall-related care provided by a general practitioner.
*Unclear*	Where authors of the systematic review have not clearly described the comparator group in included studies.
Intervention terms	
*Education*	Patient education interventions e.g., receiving information regarding falls risk and self-directed risk reduction.
*Environmental*	Home modification/equipment prescription.
*Exercise*	Movement and training focused interventions.
*Medication review*	Medication list review often coupled with deprescribing.
*Multifactorial*	Interventions which combined fall prevention strategies e.g., exercise, education, and medication review.
*Other*	Discipline specific interventions (e.g., podiatry) or medical interventions (e.g., cataract or heart surgery).
*Vitamin D*	Vitamin D prescription +/− calcium interventions.
*Quality improvement*	Interventions which sought to standardise healthcare processes (e.g., clinical pathways and staffing) in a health system.
Outcome significance	
*No difference*	Authors of meta-analyses and narrative syntheses discussed that there was no statistical difference in studies or trends observed.
*Non-significant*	Authors of meta-analyses and narrative syntheses indicated that while they did not find statistically significant changes in outcomes, they observed a trend in results.
*Significant*	Authors of meta-analyses found that the falls outcome changed in a way that was statistically significant (*p* < 0.05).

### Quality appraisal

Quality appraisal was conducted simultaneously with data extraction by five reviewers (IM, AN, PG, CM, JS) and using the Assessment of Multiple Systematic Reviews (AMSTAR)-2 tool [22]. The AMSTAR 2 tool facilitates detailed assessment of systematic reviews of randomised and non-randomised control trials, with decisions about the quality of the study made in 16 domains. Quality appraisal scores were double checked by one author (IM). Review quality was summarised into critically low, low, medium, and high quality according to scores on critical domains in the AMSTAR-2 [23]. As this umbrella review concerns only data within the included systematic review, we did not undertake quality assessment of RCTs, within systematic reviews.

### Synthesis

The results are narratively presented by fall-related outcome and then again by setting (RAC and community) where possible. Data on specific population and intervention characteristics and adverse events are also narratively synthesised. Narrative descriptions discuss the number of reviews reporting an outcome direction (improved, worsened, or no different) from the total pool of reviews which studied that outcome, intervention, and/or setting. A higher proportion of reviews finding a positive outcome with the intervention was considered a proxy for effectiveness of that intervention.

## Results

### Search strategy

**Fig. 1:**
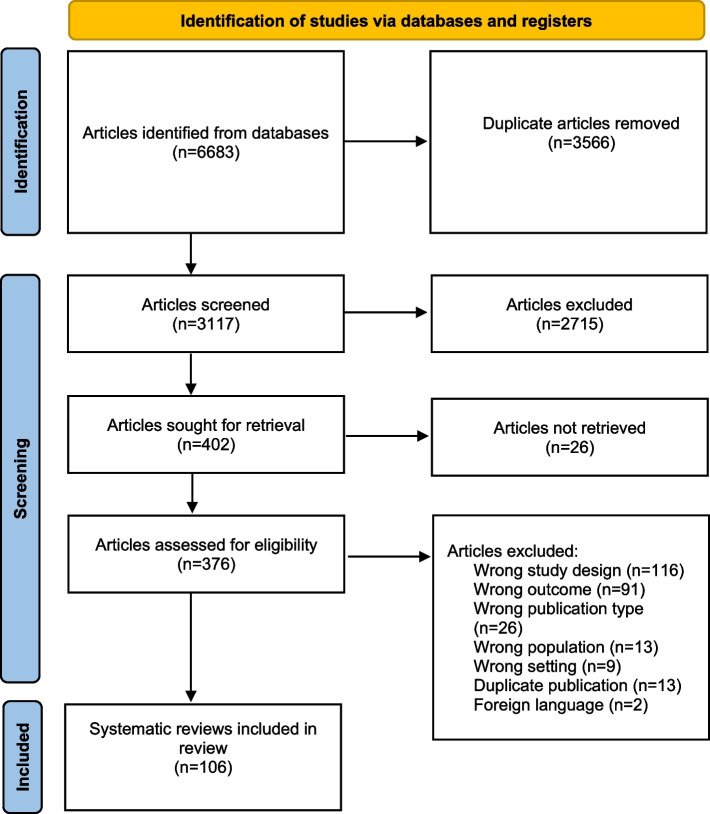
PRISMA flow diagram

The search strategy retrieved 6683 articles (CENTRAL: 102; CINAHL: 1217; EMBASE: 1712; Medline: 1433; Scopus: 1919), after removing duplicates, 3117 articles remained. Two thousand seven hundred and 41 articles were removed during title/abstract screening. A further 270 articles were removed during full text screening leaving 106 systematic reviews in this umbrella review (Fig. 1).

### Quality appraisal

Fifty-one (48%) reviews were graded as critically low quality on the AMSTAR-2 (Table 2) (Appendix 2). The remaining reviews were graded as low quality (21%, *n* = 22), moderate quality (27%, *n* = 29), and only four were graded as high quality. Common reasons for scoring poorly on the AMSTAR-2 were that the review authors did not report on funding source of included studies (94%, *n* = 100) or did not provide a list of excluded studies (79%, *n* = 84). Due to number of categories (settings, direction of outcome, and intervention type) used in this narrative synthesis it is difficult to draw attention to quality appraisal results when discussing outcomes. However, quality appraisals are discussed in-text where reviews are consistently of high or poor quality for that outcome, setting, and intervention.

**Table 2:** Characteristics of included systematic reviews (*n* = 106)*

	Number of reviews	Total^a^	Community	RAC
		106	63	19
**Interventions**	Exercise	65	38	10
	Multifactorial intervention	28	18	6
	Vitamin D	19	8	7
	Education	8	4	3
	Environmental	7	5	2
	Medication review	7	2	4
	Quality improvement	5	1	3
	Other^b^	12	9	4
**Outcomes**				
	Falls	92	51	17
	People who have had ≥1 fall	42	25	10
	Fall-related fractures	19	12	3
	Falls requiring hospitalisation	8	4	0
**AMSTAR-2**				
	Critically low	51	33	8
	Low quality	22	16	5
	Moderate quality	29	11	5
	High quality	4	3	1

*Number of reviews in each characteristic do not add to 106. ^a^ Number of reviews which were conducted in community and residential aged care = Total – (Community + RAC). ^b^ e.g., surgery, sunlight exposure, multivitamin prescription, quality improvement project such as workflow changes and introduction of guidelines

### Population

In 106 systematic reviews, sample sizes of the systematic reviews ranged from 278 to 186,932; however, 27 (25%) reviews did not present sample size information clearly. Reviews were published between 2003 and 2023. Sixty-three (60%) and 19 (15%) reviews provided fall prevention intervention outcomes in community and RAC settings respectively. The remaining reviews pooled results from community and long-term care settings (Appendix 3).

Twenty-one (20%) reviews explored population subgroups such as older and younger participants, healthy older adults, and older adults with cognitive decline. In reviews of exercise and multifactorial interventions, fall-related outcomes were either poorer [13, 24, 25] or were similar to [13, 24, 26–30] populations with more frailty, higher risk of falls, or cognitive impairment compared to more healthy counter parts. Similarly, concerning fall-related outcomes, falls and people who have had a fall, showed greater improvement [31–34] or no difference [24, 26–30, 35, 36] across various interventions among women and younger and community dwelling populations compared to older and institutionalised people.

### Intervention

Interventions explored in systematic reviews included ‘exercise’ (61%, *n* = 65), ‘multifactorial’ (26%, *n* = 28), ‘vitamin D’ (18%, *n* = 19), ‘education’ (8%, *n* = 8), ‘medication review’ (7%, *n* = 7), ‘environmental’ (7%, *n* = 7), and ‘other’ interventions (11%, *n* = 12) (Table 2). Exercise interventions often focused on Tai Chi (*n* = 11), balance (*n* = 10), resistance (*n* = 8), multicomponent exercise programs (*n* = 17) which combined types of exercise such as resistance and endurance training, or utilised technology (*n* = 9), for example, vibration (*n* = 4) or virtual reality (*n* = 1). Common components of multifactorial interventions included exercise, medication reviews or prescription, and home environment assessments. Interventions categorised as ‘other’ included cataract and heart surgeries, multivitamin prescription, nutritional supplementation, and sunlight exposure. The specific quantities of the intervention provided, for example, details of exercise/s, intensity of frequency of the intervention, or proportion of multifactorial intervention dedication to each component (e.g., 80% exercise and 20% education) were often missing from the systematic reviews.

Analyses of effectiveness by specific intervention characteristics were mixed. Reviews often reported no difference in fall-related outcomes with intervention characteristics [26], such as who led exercise interventions [35], or route of administration or adherence [37]. A dose response was proposed in several exercise and medication related reviews; for example, high vitamin D (> 800 IU per day) [32], > 3 hours of exercise per week [35], and intervention length (< 6 months and < 1 year) [32] may have a greater impact on fall incidence reduction. Supplemental components of interventions, such as the addition of calcium with ‘vitamin D’ interventions [34] and the addition of exercise and environmental interventions in multifactorial interventions [38], were sometimes discussed as demonstrating greater reduction in falls when compared to interventions which did not have these additional components.

### Comparison

While efforts were made to collect and categorise data on comparison groups (i.e., placebo, routine care, or other intervention), the comparison groups within each review were mixed or poorly described in 30% of reviews (Appendix 3). Review results were not stratified by comparator group due to the high degree of uncertainty.

### Outcomes

A total of 664 outcome results were extracted from the 106 included reviews. The incidence of falls was the most studied outcome in systematic reviews (87%, *n* = 92), followed by people who have had a fall (40%, *n* = 42), fall-related fractures (18%, *n* = 19) and falls requiring hospitalisation (8%, *n* = 8) (Table 2). The incidence of falls remained the most studied outcome in community (*n* = 51) and RAC (*n* = 17) settings.

### Falls

In all systematic reviews, the rate, risk, or number of falls were most likely to improve, either significantly or non-significantly, in ‘exercise’ (81%, *n* = 47), ‘multifactorial’ (88%, *n* = 22), and ‘vitamin D’ (69%, *n* = 13) interventions (Table 3). The rate of falls frequently did not change in reviews of ‘education’ (57%, *n* = 4), ‘environmental’ (57%, *n* = 4), and ‘medication’ (67%, *n* = 3) interventions.

**Table 3:**
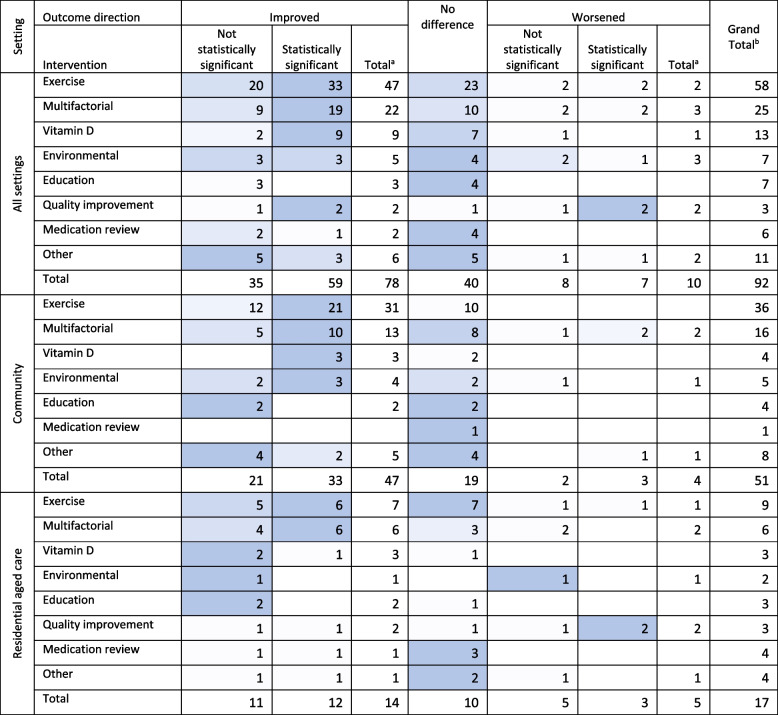
Number of systematic reviews investigating falls by intervention, setting, and direction of result

Darker shading indicates the cells with the greatest number of reviews in each row (excluding totals). ^a^ Total is the sum of unique reviews that reported an increase/decrease with the intervention. ^b^ Grand total is the sum of unique studies which explored the intervention type and setting

The type of interventions likely to impact the number, risk, or rate of falls was slightly different between RAC and community settings. Multifactorial interventions seemingly produced more consistent positive results in RAC settings. That is all six reviews reporting on ‘multifactorial’ interventions in RAC reported a statistically significant improvement in falls whereas when these interventions were implemented in community settings 62% (*n* = 10) of reviews reported a statistically significant improvement in falls. ‘vitamin D’ may be effective in community settings however the evidence is too weak to support its use in RAC settings; 75% (*n* = 3) of reviews of ‘vitamin D’ interventions in community settings reported statistically significant improvements in falls whereas in RAC settings only one review (33%) found a statistical improvement. However, all reviews of ‘vitamin D’ interventions in RAC and community settings were of low or critically low quality (Appendix 2). Lastly, reviews of ‘quality improvement’ interventions were only available in RAC settings and significantly increased the rate of falls in 66% (*n* = 2) reviews.

Ten reviews reported an increase in falls with the intervention, in seven of these reviews the increase was statistically significant. As a proportion of reviews in setting, RAC specific reviews were more likely to report an increase in falls with the intervention (29%, *n* = 5) compared to reviews of community-dwelling older adults (8%, *n* = 4). Some examples of interventions which reportedly increased falls included; ‘quality improvement’ interventions, such as staff training and education [5] and dementia care planning in RAC [13], which were associated with increased risk of falls (RR (risk ratio) 1.29, 95% CI 1.23–1.36 [5]; RaR (rate ratio) 1.84, 95% CI 1.4–2.42) [13]. The rate and likelihood of falls also increased in some exercise interventions, specifically balance, strength, and walking programs in RAC and community settings (RR 1.48, 95% CI 1.10–2.00 [39]; RaR 1.36, 95% CI 1.05–1.77) [40], an environmental intervention which introduced carpet flooring over vinyl in RAC (RaR 14.73, 95% CI 1.88–155.35) [13], a vision and hearing intervention (OR 1.7, 95% CI 1.2–2.5) [41], and in five different combinations of multifactorial interventions in community-dwelling populations [42, 43].

### People who have had one or more falls

Systematic reviews often reported falls among people who have had ≥1 fall improved, significantly or non-significantly, in most reviews of ‘exercise’ (78%, *n* = 18) and ‘multifactorial’ (73%, *n* = 14) interventions (Table 4). A third of reviews on ‘vitamin D’ (*n* = 2) and ‘education’ interventions (*n* = 1) and half of ‘quality improvement’ interventions (*n* = 2) found significant improvements in the number of people who subsequently fell. Reviews of ‘medication reviews’ (*n* = 1) found no impact on the rate of people who had a fall.

**Table 4:**
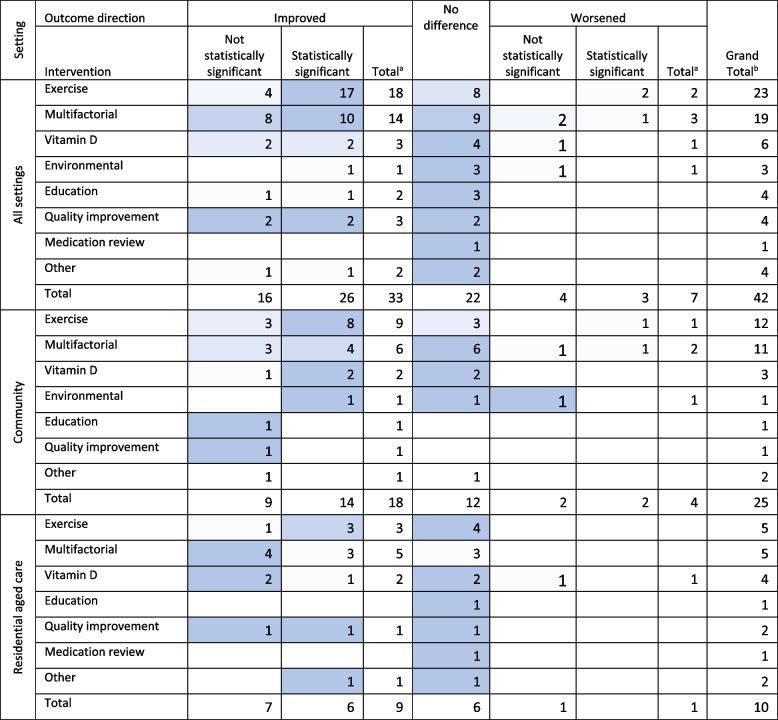
Number of systematic reviews investigating people who experienced a fall by setting, intervention, and direction of outcome

Darker shading indicates the cells with the greatest number of reviews in each row (excluding totals). ^a^ Total is the sum of unique reviews that reported an increase/decrease with the intervention. ^b^ Grand total is the sum of unique studies which explored the intervention type and setting

The profile of interventions likely to impact the number and risk of people who have had a fall differed between community and RAC settings. Overall, most reviews concluded that the number and risk of people who have had fall did not improve with any of the interventions in RAC settings; ≥ 50% of reviews reported no difference in falls in each intervention category. ‘Multifactorial’ and ‘exercise’ interventions demonstrated some promise in RAC settings (69%, *n* = 3 reviews reported a statistical improvement in people who fall). However, the reviews that reported no difference with ‘multifactorial’ or ‘exercise’ interventions in RAC were considered high or moderate quality [24, 39, 44] on the AMSTAR-2 whereas the review which found an improvement were graded as lower quality (Appendix 2). In reviews of community-dwelling older adults, ‘exercise’ interventions significantly improved the number of people who experienced ≥1 fall in 69% (*n* = 9) of reviews. ‘Vitamin D’ interventions did show some promise in community settings with 66% (*n* = 2) reporting a statistical improvement in people who had one or more falls. However, all reviews of ‘vitamin D’ interventions in community settings were of low or critically low quality [34, 45].

Seven reviews reported an increase in people who had a fall with the intervention, the increase was statistically significant in three reviews. The proportion of reviews reporting poorer outcomes was similar between RAC (10%, *n* = 1) and community settings (16%, *n* = 4). The likelihood and rate of people who experienced a fall were significantly worse in a review of multifactorial interventions (RR 1.58, 95% CI 1.01–2.48) [42] and exercise interventions which included aerobic training, balance, and cognitive components (OR 4.55, 95% CI 1.82–11.11) [36] and home exercise programs (OR 1.74, 95% CI 1.17–2.6) [46].

### Other fall-related outcomes

Reviews of exercise interventions often reported a statistically significant improvement in fall-related fractures (61%, *n* = 8) (Table 5). All other interventions including ‘multifactorial’, ‘vitamin D’, and ‘medication review’ interventions did not demonstrate an outcome trend. The results were not stratified by setting as only three of these reviews were RAC specific and 12 were community specific.

**Table 5:**
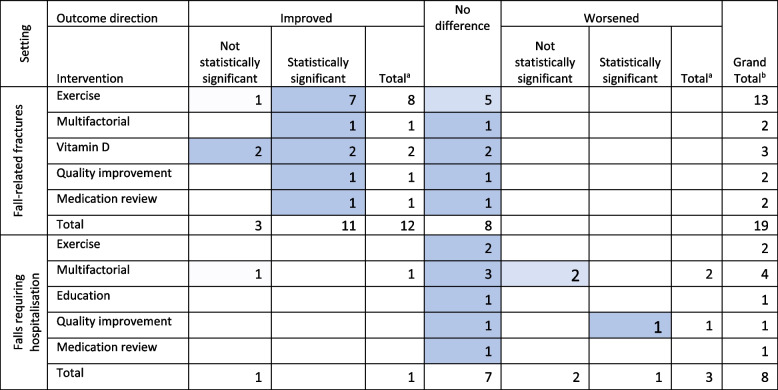
Other fall-related outcomes by intervention, outcome, and direction of outcome results

Darker shading indicates the cells with the greatest number of reviews in each row (excluding totals). ^a^ Total is the sum of unique reviews that reported an increase/decrease with the intervention. ^b^ Grand total is the sum of unique studies which explored the intervention type and setting

Falls requiring hospitalisation did not significantly improve in any of the reviews. Falls requiring hospitalisation increased with one review categorised as other; a review of quality improvement initiatives which included team changes in people < 80 years old (OR 2.79, 95% CI 1.5–5.19) [25]. No reviews of falls requiring hospitalisation were specific to RAC settings.

### Adverse events

Adverse events reported in systematic reviews were often minor and included aches, pains, and muscle soreness [13, 15, 47–49]. More severe adverse events reported in reviews included hypercalcaemia (which was higher in the intervention group in ‘vitamin D’ interventions) [34, 47, 50] and hospitalisation or medical attention (which was sometimes higher in exercise interventions) [36, 51] but not different between groups in other fall prevention intervention types [52, 53]. Death rates were also not reported to be different between intervention and control groups in reviews [47, 51–56], one review even reported it lowered in the intervention groups of deprescribing interventions (OR 0.74, 95% CI 0.65–0.84) [57].

## Discussion

In this umbrella review, ‘exercise’, ‘vitamin D’, and ‘multifactorial’ interventions were found to be the most studied intervention types in gold standard evidence (reviews of RCTs) and had some promising results. Reviews of ‘exercise’ interventions reported statistically significant improvements in falls, the number of people who had ≥1 fall and fall-related fractures more consistently than reviews of other intervention types across all settings. The direction of and consistency of outcomes (positive/negative) reported in systematic reviews was often consistent between RAC and community settings. Notable exceptions were ‘vitamin D’ interventions, which demonstrated consistent improvements in falls and people who had a fall in community settings but no difference in RAC, and ‘multifactorial’ interventions which may be deliver more consistent outcomes in RAC settings rather than community.

The use of ‘vitamin D’ is recommended as routine care to prevent the incidence of falls and promote bone health in local guidelines for all RAC dwelling older adults [6, 58]. However, in this umbrella review, we found weak evidence to support its use in RAC. We have three hypotheses to explain the difference in outcome trends with ‘vitamin D’ between settings. First, RAC residents are frequently frailer than community-dwelling older adults. In practice this could mean that ‘vitamin D’ interventions are not sufficient to counter the extreme risk of falling in this population. Instead, if ‘vitamin D’ interventions are appropriate in RAC, they may be more effective as part of a larger ‘multifactorial’ intervention. Second, the few studies that examined ‘vitamin D’ interventions in RAC may not have been appropriately designed. The result may have been confounded by usual care, which increasingly includes vitamin D prescription [59], and is poorly described in included reviews. It is also possible that ‘vitamin D’ interventions may be beneficial for only a subgroup of the RAC population; for example, in community settings ‘Vitamin D’ interventions have a greater impact on falls reduction in people who are deficient in Vitamin D at baseline [16]. However, no reviews of vitamin D in RAC included a population subgroup analysis. Additionally, and more broadly, some of the fall’s outcomes used to measure change (i.e., people who had a fall) in RAC setting reviews may not be appropriate as they are less sensitive to change in a population who falls frequently. Further research is required to confirm the role of ‘vitamin D’ for fall prevention interventions in RAC in the future. For now, our finding should not dissuade the use of vitamin D supplementation in RAC to maintain adequate nutrition and bone health.

No intervention type was shown to be effective at improving fall-related outcomes in all systematic reviews. For example, reviews of ‘multifactorial’ interventions in RAC settings which frequently significantly reduced falls (100%, *n* = 6) also reported increased falls (33%, *n* = 2) or made no difference (50%, *n* = 3) with different combinations of the ‘multifactorial’ intervention. This result in our review may be because fall contributing factors are likely to vary significantly by individual and environmental factors. Therefore, a blanket intervention across a population only has a marginal effect as it does not address the specific underlying and individual key contributing factors. In our review, some interventions may have demonstrated more consistent positive trends in their results as they are simply appropriate for most of the population. For example, ‘exercise’ may be broadly beneficial as overall the older adults in both RAC and community settings typically do not meet their physical activity requirements [60, 61]. The results of this review make it difficult to provide clear applicable recommendations for practice.

‘Quality improvement’ interventions were highlighted as potentially harmful in this review. More than 50% of reviews reported an increase in the rate of falls with ‘quality improvement’ interventions in RAC and community settings. However, this finding needs to be interpreted with caution. This result is based on a limited number of underlying RCTs which were rated as low quality by included reviews [13]. Additionally, ‘quality improvement’ interventions included in this review, such as staff education and implementation of a new care pathway, may reflect an increase in the rate of incident reporting rather than true increase in the number of falls.

The clearest recommendation we can make based on this review is that ‘exercise’ is likely the most beneficial component of a falls prevention plan for older adults living in the community and RAC. In resource constrained environments, ‘exercise’ should be a minimum, blanket intervention for falls prevention in older adults [28]. The addition of other strategies to make the intervention ‘multifactorial’ may also be beneficial in both settings. These results suggest, from a provider’s perspective who delivers care in both RAC and community settings, that similar fall prevention interventions are effective and could be delivered simultaneously across the care spectrum. Simultaneous application of fall prevention interventions could leverage provide buy-in and resources and reduce complexity. However, it is important to recognise that community and RAC settings are distinctively different. In practice, careful implementation planning, which includes evaluating client, environmental, and service falls risk factors, and tailoring interventions will still be required to adapt the intervention to the setting and client needs.

## Limitations

This umbrella review does not follow a reporting standard guideline (e.g., preferred reporting items for systematic review and meta-analyses (PRISMA)) [62] as in 2021 no reporting standards were available for umbrella reviews. Our review is also limited as it does not account for multiple entries of the same RCT across the 106 reviews. The primary study overlap may have introduced bias toward the population, intervention, and outcomes of certain publications. Our results may also over-represent the change in outcomes due to the inclusion of non-statistically significant results as they were categorised as improved or worsened based off review authors subjective description. However, this method was chosen to represent the results from reviews with a narrative synthesis and fall prevention interventions which may not yet have sufficient data to produce a meta-analysis. Lastly, our review does not reflect on the effect size as the review did not include individual RCT data and meta-analysis data had primary study overlap and was inconsistently calculated.

## Conclusion

In this review outcomes achieved with fall prevention intervention types were often similar across RAC and community settings. ‘Exercise’ interventions are the most likely to improve fall outcomes, rate of falls, and number of people who experience a fall, in both community and RAC populations compared to other intervention types. ‘Exercise’ interventions should be an essential component of service level fall prevention programs for older adults in any setting. Augmenting ‘exercise’ interventions to create ‘multifactorial’ interventions is also likely to reduce the incidence of falls in both community and RAC. However, the specific components of a ‘multifactorial’ intervention likely need to be tailored to each older adults fall risk factors irrespective of their setting.

### Supplementary Information

**Additional file 1.**

## Data Availability

All data generated or analysed during this study are included in this published article and its supplementary information files.

## Abbreviations

AMSTAR: Assessment of multiple systematic reviews
PRISMA: Preferred reporting items for systematic review and meta-analyses
RAC: Residential aged care
RaR: Rate ratio
RCT: Randomised control trial
RR: Risk ratio
