# Supplementary material for: Effectiveness of fall prevention interventions in residential aged care and community settings: an umbrella review
Source: BMC Geriatr. 2024 Jan 19;24:75. doi: 10.1186/s12877-023-04624-4 (PMC10799511; doi:10.1186/s12877-023-04624-4)
Supplement: Supplementary file 1 — Additional file 1. [file 12877_2023_4624_MOESM1_ESM.docx]

# Appendix 1

**Table 1. Medline Ovid search strategy**

| 1 | Accidental Falls/ |
| --- | --- |
| 2 | (fall or falls or faller$ or fall-related).tw. |
| 3 | exp Aged/ |
| 4 | (old$ or senior$ or elderly or aged).tw. |
| 5 | 3 or 4 |
| 6 | (systematic review or meta-analysis).pt. |
| 7 | (systematic review or meta-analysis).tw. |
| 8 | 6 or 7 |
| 9 | 1 or 2 |
| 10 | 5 and 8 and 9 |
| 11 | limit 10 to (english language and yr="2000 -Current") |

**Table 2. EMBASE search strategy**

| 1 | falling/ |
| --- | --- |
| 2 | (fall or falls or faller$ or fall-related).tw. |
| 3 | exp Aged/ |
| 4 | (old$ or senior$ or elderly or aged).tw. |
| 5 | (systematic review or meta-analysis).tw. |
| 6 | 3 or 4 |
| 7 | 1 or 2 |
| 8 | 5 and 6 and 7 |
| 9 | limit 8 to (english language and yr="2000 -Current") |

**Table 3. Scopus search strategy**

| ( TITLE-ABS-KEY ( ( fall OR falls OR faller* OR fall-related ) ) AND TITLE-ABS-KEY ( old* OR senior* OR elderly OR aged ) AND TITLE-ABS-KEY ( systematic AND review OR meta-analysis ) ) AND PUBYEAR > 1999 AND ( LIMIT-TO ( LANGUAGE , "English" ) ) |
| --- |

**Table 4. CENTRAL search strategy**

| #1 | (fall or falls or faller* or fall-related):ti,ab,kw |
| --- | --- |
| #2 | MeSH descriptor: [Accidental Falls] explode all trees |
| #3 | (old* or senior* or elderly or aged):ti,ab,kw |
| #4 | MeSH descriptor: [Aged] explode all trees |
| #5 | (systematic review or meta-analysis):ti,ab,kw |
| #6 | #1 OR #2 |
| #7 | #3 OR #4 |
| #8 | #6 AND #7 AND #5 in Cochrane Reviews limit date |

**Table 5. CINAHL search strategy**

| S1 | MH "Accidental Falls" |
| --- | --- |
| S2 | TI ( fall or falls or faller* or fall-related ) OR AB ( fall or falls or faller* or fall-related ) |
| S3 | (MH "Aged+") |
| S4 | TI ( old* or senior* or elderly or aged ) OR AB ( old* or senior* or elderly or aged ) |
| S5 | (MH "Systematic Review") OR (MH "Meta Analysis") |
| S6 | TI ( systematic review or meta-analysis ) OR AB ( systematic review or meta-analysis ) |
| S7 | S1 OR S2 |
| S8 | S3 OR S4 |
| S9 | S5 OR S6 |
| S10 | S7 AND S8 AND S9  Published Date: 20000101-20211231; English Language |

# Appendix 2 AMSTAR-2 quality rating

**Table 1.** AMSTAR-2 results

|  |  |  |  |  |  |  |  |  |  |  |  |  |  | **Key:** | |  | **Yes** |
| --- | --- | --- | --- | --- | --- | --- | --- | --- | --- | --- | --- | --- | --- | --- | --- | --- | --- |
|  |  |  |  |  |  |  |  |  |  |  |  |  |  |  |  |  | **Not applicable** |
|  |  |  |  |  |  |  |  |  |  |  |  |  |  |  |  |  | **No** |
| **Reference** | **item 1** | **item 2** | **item 3** | **Item 4** | **item 5** | **Item 6** | **Item 7** | **Item 8** | **Item 9** | **Item 10** | **Item 11** | **Item 12** | **Item 13** | **Item 14** | **Item 15** | **Item 16** | **AMSTAR-2 quality category** |
| [1] |  |  |  |  |  |  |  |  |  |  |  |  |  |  |  |  | Low |
| [2] |  |  |  |  |  |  |  |  |  |  |  |  |  |  |  |  | Critically Low |
| [3] |  |  |  |  |  |  |  |  |  |  |  |  |  |  |  |  | Moderate |
| [4] |  |  |  |  |  |  |  |  |  |  |  |  |  |  |  |  | Moderate |
| [5] |  |  |  |  |  |  |  |  |  |  |  |  |  |  |  |  | Critically Low |
| [6] |  |  |  |  |  |  |  |  |  |  |  |  |  |  |  |  | Moderate |
| [7] |  |  |  |  |  |  |  |  |  |  |  |  |  |  |  |  | Moderate |
| [8] |  |  |  |  |  |  |  |  |  |  |  |  |  |  |  |  | Moderate |
| [9] |  |  |  |  |  |  |  |  |  |  |  |  |  |  |  |  | Low |
| [10] |  |  |  |  |  |  |  |  |  |  |  |  |  |  |  |  | Critically Low |
| [11] |  |  |  |  |  |  |  |  |  |  |  |  |  |  |  |  | Low |
| [12] |  |  |  |  |  |  |  |  |  |  |  |  |  |  |  |  | High |
| [13] |  |  |  |  |  |  |  |  |  |  |  |  |  |  |  |  | Critically Low |
| [14] |  |  |  |  |  |  |  |  |  |  |  |  |  |  |  |  | Moderate |
| [15] |  |  |  |  |  |  |  |  |  |  |  |  |  |  |  |  | Critically Low |
| [16] |  |  |  |  |  |  |  |  |  |  |  |  |  |  |  |  | Moderate |
| [17] |  |  |  |  |  |  |  |  |  |  |  |  |  |  |  |  | Low |
| [18] |  |  |  |  |  |  |  |  |  |  |  |  |  |  |  |  | Critically Low |
| [19] |  |  |  |  |  |  |  |  |  |  |  |  |  |  |  |  | Critically Low |
| [20] |  |  |  |  |  |  |  |  |  |  |  |  |  |  |  |  | Moderate |
| [21] |  |  |  |  |  |  |  |  |  |  |  |  |  |  |  |  | Critically Low |
| [22] |  |  |  |  |  |  |  |  |  |  |  |  |  |  |  |  | Moderate |
| [23] |  |  |  |  |  |  |  |  |  |  |  |  |  |  |  |  | Moderate |
| [24] |  |  |  |  |  |  |  |  |  |  |  |  |  |  |  |  | Critically Low |
| [25] |  |  |  |  |  |  |  |  |  |  |  |  |  |  |  |  | Moderate |
| [26] |  |  |  |  |  |  |  |  |  |  |  |  |  |  |  |  | Moderate |
| [27] |  |  |  |  |  |  |  |  |  |  |  |  |  |  |  |  | Low |
| [28] |  |  |  |  |  |  |  |  |  |  |  |  |  |  |  |  | Moderate |
| [29] |  |  |  |  |  |  |  |  |  |  |  |  |  |  |  |  | Critically Low |
| [30] |  |  |  |  |  |  |  |  |  |  |  |  |  |  |  |  | Low |
| [31] |  |  |  |  |  |  |  |  |  |  |  |  |  |  |  |  | Critically Low |
| [32] |  |  |  |  |  |  |  |  |  |  |  |  |  |  |  |  | Critically Low |
| [33] |  |  |  |  |  |  |  |  |  |  |  |  |  |  |  |  | Critically Low |
| [34] |  |  |  |  |  |  |  |  |  |  |  |  |  |  |  |  | Critically Low |
| [35] |  |  |  |  |  |  |  |  |  |  |  |  |  |  |  |  | Critically Low |
| [36] |  |  |  |  |  |  |  |  |  |  |  |  |  |  |  |  | Low |
| [37] |  |  |  |  |  |  |  |  |  |  |  |  |  |  |  |  | Critically Low |
| [38] |  |  |  |  |  |  |  |  |  |  |  |  |  |  |  |  | Critically Low |
| [39] |  |  |  |  |  |  |  |  |  |  |  |  |  |  |  |  | Low |
| [40] |  |  |  |  |  |  |  |  |  |  |  |  |  |  |  |  | Low |
| [41] |  |  |  |  |  |  |  |  |  |  |  |  |  |  |  |  | Critically Low |
| [42] |  |  |  |  |  |  |  |  |  |  |  |  |  |  |  |  | Critically Low |
| [43] |  |  |  |  |  |  |  |  |  |  |  |  |  |  |  |  | Critically Low |
| [44] |  |  |  |  |  |  |  |  |  |  |  |  |  |  |  |  | Critically Low |
| [45] |  |  |  |  |  |  |  |  |  |  |  |  |  |  |  |  | High |
| [46] |  |  |  |  |  |  |  |  |  |  |  |  |  |  |  |  | Low |
| [47] |  |  |  |  |  |  |  |  |  |  |  |  |  |  |  |  | Low |
| [48] |  |  |  |  |  |  |  |  |  |  |  |  |  |  |  |  | Moderate |
| [49] |  |  |  |  |  |  |  |  |  |  |  |  |  |  |  |  | Low |
| [50] |  |  |  |  |  |  |  |  |  |  |  |  |  |  |  |  | Critically Low |
| [51] |  |  |  |  |  |  |  |  |  |  |  |  |  |  |  |  | Moderate |
| [52] |  |  |  |  |  |  |  |  |  |  |  |  |  |  |  |  | Critically Low |
| [53] |  |  |  |  |  |  |  |  |  |  |  |  |  |  |  |  | Low |
| [54] |  |  |  |  |  |  |  |  |  |  |  |  |  |  |  |  | Critically Low |
| [55] |  |  |  |  |  |  |  |  |  |  |  |  |  |  |  |  | Low |
| [56] |  |  |  |  |  |  |  |  |  |  |  |  |  |  |  |  | Moderate |
| [57] |  |  |  |  |  |  |  |  |  |  |  |  |  |  |  |  | Critically Low |
| [58] |  |  |  |  |  |  |  |  |  |  |  |  |  |  |  |  | Critically Low |
| [59] |  |  |  |  |  |  |  |  |  |  |  |  |  |  |  |  | Critically Low |
| [60] |  |  |  |  |  |  |  |  |  |  |  |  |  |  |  |  | Moderate |
| [61] |  |  |  |  |  |  |  |  |  |  |  |  |  |  |  |  | Critically Low |
| [62] |  |  |  |  |  |  |  |  |  |  |  |  |  |  |  |  | Low |
| [63] |  |  |  |  |  |  |  |  |  |  |  |  |  |  |  |  | Critically Low |
| [64] |  |  |  |  |  |  |  |  |  |  |  |  |  |  |  |  | Critically Low |
| [65] |  |  |  |  |  |  |  |  |  |  |  |  |  |  |  |  | Critically Low |
| [66] |  |  |  |  |  |  |  |  |  |  |  |  |  |  |  |  | Critically Low |
| [67] |  |  |  |  |  |  |  |  |  |  |  |  |  |  |  |  | Critically Low |
| [68] |  |  |  |  |  |  |  |  |  |  |  |  |  |  |  |  | Moderate |
| [69] |  |  |  |  |  |  |  |  |  |  |  |  |  |  |  |  | Low |
| [70] |  |  |  |  |  |  |  |  |  |  |  |  |  |  |  |  | Low |
| [71] |  |  |  |  |  |  |  |  |  |  |  |  |  |  |  |  | Low |
| [72] |  |  |  |  |  |  |  |  |  |  |  |  |  |  |  |  | Moderate |
| [73] |  |  |  |  |  |  |  |  |  |  |  |  |  |  |  |  | Critically Low |
| [74] |  |  |  |  |  |  |  |  |  |  |  |  |  |  |  |  | Low |
| [75] |  |  |  |  |  |  |  |  |  |  |  |  |  |  |  |  | Critically Low |
| [76] |  |  |  |  |  |  |  |  |  |  |  |  |  |  |  |  | Moderate |
| [77] |  |  |  |  |  |  |  |  |  |  |  |  |  |  |  |  | Moderate |
| [78] |  |  |  |  |  |  |  |  |  |  |  |  |  |  |  |  | Critically Low |
| [79] |  |  |  |  |  |  |  |  |  |  |  |  |  |  |  |  | Critically Low |
| [80] |  |  |  |  |  |  |  |  |  |  |  |  |  |  |  |  | Critically Low |
| [81] |  |  |  |  |  |  |  |  |  |  |  |  |  |  |  |  | Moderate |
| [82] |  |  |  |  |  |  |  |  |  |  |  |  |  |  |  |  | Moderate |
| [83] |  |  |  |  |  |  |  |  |  |  |  |  |  |  |  |  | Critically Low |
| [84] |  |  |  |  |  |  |  |  |  |  |  |  |  |  |  |  | Critically Low |
| [85] |  |  |  |  |  |  |  |  |  |  |  |  |  |  |  |  | Moderate |
| [86] |  |  |  |  |  |  |  |  |  |  |  |  |  |  |  |  | Low |
| [87] |  |  |  |  |  |  |  |  |  |  |  |  |  |  |  |  | Low |
| [88] |  |  |  |  |  |  |  |  |  |  |  |  |  |  |  |  | Critically Low |
| [89] |  |  |  |  |  |  |  |  |  |  |  |  |  |  |  |  | Critically Low |
| [90] |  |  |  |  |  |  |  |  |  |  |  |  |  |  |  |  | Critically Low |
| [91] |  |  |  |  |  |  |  |  |  |  |  |  |  |  |  |  | Low |
| [92] |  |  |  |  |  |  |  |  |  |  |  |  |  |  |  |  | Critically Low |
| [93] |  |  |  |  |  |  |  |  |  |  |  |  |  |  |  |  | Moderate |
| [94] |  |  |  |  |  |  |  |  |  |  |  |  |  |  |  |  | Critically Low |
| [95] |  |  |  |  |  |  |  |  |  |  |  |  |  |  |  |  | Critically Low |
| [96] |  |  |  |  |  |  |  |  |  |  |  |  |  |  |  |  | Critically Low |
| [97] |  |  |  |  |  |  |  |  |  |  |  |  |  |  |  |  | Critically Low |
| [98] |  |  |  |  |  |  |  |  |  |  |  |  |  |  |  |  | High |
| [99] |  |  |  |  |  |  |  |  |  |  |  |  |  |  |  |  | Critically Low |
| [100] |  |  |  |  |  |  |  |  |  |  |  |  |  |  |  |  | High |
| [101] |  |  |  |  |  |  |  |  |  |  |  |  |  |  |  |  | Moderate |
| [102] |  |  |  |  |  |  |  |  |  |  |  |  |  |  |  |  | Moderate |
| [103] |  |  |  |  |  |  |  |  |  |  |  |  |  |  |  |  | Moderate |
| [104] |  |  |  |  |  |  |  |  |  |  |  |  |  |  |  |  | Moderate |
| [105] |  |  |  |  |  |  |  |  |  |  |  |  |  |  |  |  | Critically Low |
| [106] |  |  |  |  |  |  |  |  |  |  |  |  |  |  |  |  | Critically Low |

**Item 1:** Did the research questions and inclusion criteria for the review include the components of PICO?; **Item 2:** Did the report of the review contain an explicit statement that the review methods were established prior to conduct of the review and did the report justify any significant deviations from the protocol?; **Item 3:** Did the review authors explain their selection of the study designs for inclusion in the review?; **Item 4:** Did the review authors use a comprehensive literature search strategy?; **Item 5:** Did the review authors perform study selection in duplicate?; **Item 6:** Did the review authors perform data extraction in duplicate?; **Item 7:** Did the review authors provide a list of excluded studies and justify the exclusions?; **Item 8:** Did the review authors describe the included studies in adequate detail?; **Item 9:** Did the review authors use a satisfactory technique for assessing the risk of bias (RoB) in individual studies that were included in the review?; **Item 10:** Did the review authors report on the sources of funding for the studies included in the review?; **Item 11:** If meta-analysis was justified did the review authors use appropriate methods for statistical combination of results? (Only complete this item if meta-analysis of other data synthesis techniques were reported); **Item 12:** If meta-analysis was performed did the review authors assess the potential impact of RoB in individual studies on the results of the meta-analysis or other evidence synthesis?; **Item 13:** Did the review authors account for RoB in individual studies when interpreting/discussing the results of the review?; **Item 14:** Did the review authors provide a satisfactory explanation for, and discussion of, any heterogeneity observed in the results of the review?; **Item 15:** If they performed quantitative synthesis did the review authors carry out an adequate investigation of publication bias (small study bias) and discuss its likely impact on the results of the review?; **Item 16:** Did the review authors report any potential sources of conflict of interest, including any funding they received for conducting the review?

**Table 2.** Number of reviews in all settings in each AMSTAR rating category by outcome

| Outcome | Intervention | Final AMSTAR quality | | | | |
| --- | --- | --- | --- | --- | --- | --- |
|  |  | Critically Low | Low | Moderate | High | Grand Total^a^ |
| Falls | Exercise | 33 | 8 | 16 | 1 | 57 |
|  | Multifactorial | 12 | 6 | 4 | 3 | 25 |
|  | Vitamin D | 6 | 3 | 4 |  | 13 |
|  | Environmental | 4 |  | 1 | 2 | 7 |
|  | Education | 4 |  | 1 | 2 | 7 |
|  | Quality improvement | 1 |  | 1 | 1 | 3 |
|  | Medication review | 2 | 2 | 1 | 1 | 6 |
|  | Other | 7 | 2 | 1 | 1 | 11 |
| People who had a fall | Exercise | 10 | 5 | 7 | 1 | 23 |
|  | Multifactorial | 8 | 5 | 4 | 2 | 19 |
|  | Vitamin D | 3 | 2 |  | 1 | 6 |
|  | Environmental | 1 |  |  | 2 | 3 |
|  | Education | 1 |  | 1 | 2 | 4 |
|  | Quality improvement | 1 |  | 2 | 1 | 4 |
|  | Medication review |  |  |  | 1 | 1 |
|  | Other | 1 | 2 |  | 1 | 4 |
| Fall-related fractures | Exercise | 7 | 1 | 3 | 2 | 13 |
|  | Multifactorial | 1 | 1 |  |  | 2 |
|  | Vitamin D | 2 |  | 1 |  | 3 |
|  | Quality improvement |  |  | 1 | 1 | 2 |
|  | Medication review |  |  | 1 | 1 | 2 |
| Number of falls requiring hospitalisation | Exercise | 1 |  | 1 |  | 2 |
|  | Multifactorial |  | 2 | 1 | 1 | 4 |
|  | Education |  |  | 1 |  | 1 |
|  | Quality improvement |  |  | 1 |  | 1 |
|  | Medication review |  |  | 1 |  | 1 |
| Grand Total | | 51 | 22 | 28 | 4 | 106 |

Darker shading indicates the cells with the greatest number of reviews in each row (excluding totals). ^a^ Grand total is the sum of unique studies which explored the intervention type and setting.

**Table 3.** Number of community-based reviews in each AMSTAR rating category by outcome

|  |  | Final AMSTAR quality | | | | |
| --- | --- | --- | --- | --- | --- | --- |
| Outcome | Intervention | Critically Low | Low | Moderate | High | Grand Total^a^ |
| Falls | Exercise | 21 | 6 | 7 |  | 34 |
|  | Multifactorial | 7 | 4 | 3 | 2 | 16 |
|  | Vitamin D | 2 | 2 |  |  | 4 |
|  | Environmental | 3 |  | 1 | 1 | 5 |
|  | Education | 3 |  |  | 1 | 4 |
|  | Medication review | 1 |  |  |  | 1 |
|  | Other | 6 | 2 |  |  | 8 |
| People who had a fall | Exercise | 6 | 4 | 2 |  | 12 |
|  | Multifactorial | 4 | 4 | 2 | 1 | 11 |
|  | Vitamin D | 1 | 2 |  |  | 3 |
|  | Environmental |  |  |  | 1 | 1 |
|  | Education |  |  |  | 1 | 1 |
|  | Quality improvement |  |  | 1 |  | 1 |
|  | Other |  | 2 |  |  | 2 |
| Fall-related fractures | Exercise | 5 | 1 | 1 | 1 | 8 |
|  | Multifactorial |  | 1 |  |  | 1 |
|  | Vitamin D | 2 |  |  |  | 2 |
|  | Quality improvement |  |  | 1 |  | 1 |
|  | Medication review |  |  | 1 |  | 1 |
| Falls requiring hospitalisation | Multifactorial |  | 2 |  | 1 | 3 |
|  | Medication review |  |  | 1 |  | 1 |
| Grand Total | | 33 | 16 | 11 | 3 | 63 |

Darker shading indicates the cells with the greatest number of reviews in each row (excluding totals). ^a^ Grand total is the sum of unique studies which explored the intervention type and setting.

**Table 4.** Number of residential aged care -based reviews in each AMSTAR rating category by outcome

|  |  | Final AMSTAR quality | | | | |
| --- | --- | --- | --- | --- | --- | --- |
| Outcome | Intervention | Critically Low | Low | Moderate | High | Grand Total^a^ |
| Falls | Exercise | 4 | 1 | 3 | 1 | 9 |
|  | Multifactorial | 3 | 1 | 1 | 1 | 6 |
|  | Vitamin D | 2 | 1 |  |  | 3 |
|  | Environmental | 1 |  |  | 1 | 2 |
|  | Education | 1 |  | 1 | 1 | 3 |
|  | Quality improvement | 1 |  | 1 | 1 | 3 |
|  | Medication review | 1 | 1 | 1 | 1 | 4 |
|  | Other | 2 |  | 1 | 1 | 4 |
| People who had a fall | Exercise | 2 | 1 | 2 | 1 | 6 |
|  | Multifactorial | 2 | 1 | 1 | 1 | 5 |
|  | Vitamin D | 2 | 1 |  | 1 | 4 |
|  | Education |  |  |  | 1 | 1 |
|  | Quality improvement | 1 |  |  | 1 | 2 |
|  | Medication review |  |  |  | 1 | 1 |
|  | Other | 1 |  |  | 1 | 2 |
| Fall-related fractures | Exercise | 1 |  |  | 1 | 2 |
|  | Vitamin D | 1 |  |  |  | 1 |
|  | Quality improvement |  |  |  | 1 | 1 |
|  | Medication review |  |  |  | 1 | 1 |
| Grand Total | | 8 | 5 | 5 | 1 | 19 |

Darker shading indicates the cells with the greatest number of reviews in each row (excluding totals). ^a^ Grand total is the sum of unique studies which explored the intervention type and setting.

# Appendix 3

**Table 1. Summary and AMSTAR Results of Included studies**

| **Review** | **N studies included** | **Settings** | **Population*** | **Intervention** | **Comparison group** | **Outcomes extracted** |
| --- | --- | --- | --- | --- | --- | --- |
| Almutairi 2020 [1] | 25 | RAC |  | Medication review | Unclear | Falls |
| Arnold 2008 [2] | 22 | Community |  | Exercise | Passive | Falls |
| Barker 2015 [3] | 6 | RAC |  | Exercise | Passive | Falls |
| Barreto 2021 [4] | 7 | Community & RAC |  | Exercise | Passive | Falls |
| Beswick 2008 [5] | 89 | Community |  | Multifactorial intervention | Passive | Falls |
| Bierma-Zeinstra 2010 [6] | 9 | Community |  | Exercise | Passive | Falls |
| Bischoff-Ferrari 2009 [7] | 8 | Community & RAC |  | Vitamin D | Passive | Falls |
| Bischoff-Ferrari 2004 [8] | 5 | Community & RAC |  | Vitamin D | Active | Falls |
| Boll et al, 2014 [9] | 20 | Community & RAC |  | Vitamin D | Active | Falls |
| Bonner 2021 [10] | 4 | Community |  | Other | Passive | Falls |
| Cadore 2013 [11] | 20 | Community |  | Exercise | Passive | Falls |
| Cameron 2018 [12] | 95 | RAC |  | Exercise; Medication review; Vitamin D; Other; Education; Quality improvement; Multifactorial interventions; Environmental | Passive; Active; Unclear | Falls; Fallers; Fall-related fractures |
| Campbell 2007 [13] | 14 | Community |  | Exercise | Unclear | Falls |
| Cao 2018 [14] | 9 | RAC |  | Exercise | Passive | Falls |
| Cao 2022 [99] | 12 | Community |  | Exercise | Passive | Falls, Fallers |
| Caristia 2021 [15] | 32 | Community | Healthy | Exercise | Unclear | Falls; Fallers; Fall-related fractures |
| Chamberlain-Carter 2021 [16] | 11 | Community | Parkinson’s disease | Exercise | Passive | Falls |
| Chan 2021 [17] | 31 | Community |  | Other | Passive | Fallers |
| Chan 2015 [18] | 7 | Community |  | Exercise | Passive | Falls |
| Cheng 2018 [19] | 49 | Community |  | Multifactorial intervention; Other | Unclear | Falls |
| Choi 2012 [20] | 17 | Community |  | Multifactorial intervention; Other | Unclear | Falls |
| Chua 2011 [21] | 4 | RAC |  | Vitamin D | Active | Falls; Fallers |
| Claudino 2021 [22] | 5 | Community |  | Exercise | Active | Falls |
| Clemson 2008 [23] | 6 | Community |  | Environmental | Unclear | Falls |
| Clemson 2023 [100] | 22 | Community |  | Environmental; Behavioural strategies; Education; Multifactorial interventions | Passive | Falls; Fallers |
| Coussement 2008 [24] | 8 | RAC & Hospital |  | Multifactorial intervention | Unclear | Falls; Fallers |
| Dautzenberg 2021 [25] | 192 | Community |  | Mixed; Quality improvement; Exercise; Multifactorial intervention; | Passive | Falls; Fallers; Fall-related fractures |
| De Kam 2009 [26] | 28 | Community & RAC | Low bone density | Exercise | Unclear | Falls |
| Desapriya 2010 [27] | 3 | Community |  | Other | Active | Falls |
| Devasahayam 2023 [101] | 29 | Community & RAC |  | Exercise |  | Falls |
| Fernandez -Rodriguez 2021 [28] | 39 | Community |  | Exercise | Unclear | Falls |
| Fisseha 2017 [29] | 5 | Community |  | Exercise | Passive | Falls |
| Francis-Coad 2018 [30] | 12 | RAC |  | Multifactorial intervention; | Unclear | Falls; Fallers |
| Garcia-Hermoso 2020 [31] | 90 | Community & RAC |  | Exercise | Unclear | Falls; Fall-related fractures; Falls requiring hospitalisation |
| Garcia-Lopez 2021 [32] | 7 | Community & RAC | Parkinson’s disease | Exercise | Active; Unclear | Falls |
| Gates 2008 [33] | 19 | Community | High risk of falls | Mixed | Unclear | Fallers |
| Gomersall 2020 [34] | 5 | Community | High risk of falls | Exercise | Unclear | Falls |
| Goodwin 2014 [35] | 17 | Community & RAC | High risk of falls | Multifactorial intervention | Unclear | Falls; Fall-related fractures |
| Guirguis-Blake 2018 [36] | 62 | Community | High risk of falls | Multifactorial intervention; Exercise; Vitamin D | Passive | Falls; Fallers |
| Gulka 2020 [37] | 36 | RAC |  | Mixed; Other; Medication review; Exercise; Multifactorial intervention | Unclear | Falls; Fallers |
| Guo 2014 [38] | 111 | Community & RAC |  | Exercise; Other; Vitamin D; Environmental; Other; Education; Multifactorial intervention; | Passive | Falls |
| Gutiérrez-Robledo 2021 [39] | 8 | Community | Cataract surgery | Other | Passive | Fallers |
| Haines 2018 [40] | 16 | Community | Recently discharged from hospital | Exercise | Passive | Falls; Fallers |
| Harling 2008 [41] | 7 | Community & RAC |  | Exercise | Unclear | Falls |
| Herbert 2008 [42] | 44 | Community & RAC |  | Exercise | Unclear | Falls |
| Hill 2018 [43] | 26 | Community | Asian | Vitamin D; Environmental; Education; Mixed; Multifactorial intervention; Exercise | Unclear | Falls; Fall-related fractures |
| Hill 2015 [44] | 12 | Community |  | Exercise | Unclear | Falls; Fallers; Fall-related fractures |
| Hopewell 2018 [45] | 62 | Community |  | Multifactorial interventions | Passive; Active | Falls; Fallers; Falls requiring hospitalisation |
| Hopewell 2019 [46] | 41 | Community |  | Multifactorial interventions | Passive | Falls; Fallers; Fall-related fractures; Falls requiring hospitalisation |
| Huang 2017 [107] | 18 | Community |  | Exercise | Passive | Falls; Fallers |
| Jehu 2023 [102] | 12 | Community & RAC |  | Exercise |  | Falls |
| Jepsen 2017 [48] | 14 | Community & RAC |  | Exercise | Active | Falls; Fallers |
| Kalyani 2010 [49] | 10 | Community & RAC |  | Vitamin D | Active | Fallers |
| Keay 2018 [50] | 7 | Community | Visual impairment | Exercise | Passive | Falls |
| Kong 2022 [103] | 32 | Community & RAC |  | Vitamin D |  | Falls |
| Kua 2019 [51] | 41 | RAC |  | Medication review; Education | Active | Falls |
| Latham 2003 [52] | 13 | Community & RAC |  | Vitamin D | Passive | Falls |
| Lee 2021 [53] | 5 | Community & RAC |  | Medication review | Passive | Falls |
| Lee 2017 [54] | 21 | RAC |  | Exercise | Passive; Active | Falls; Fallers |
| Lee 2020 [55] | 46 | Community |  | Multifactorial intervention | Passive | Falls; Fallers |
| Li 2021 [56] | 9 | Community | Cognitive impairment | Multifactorial intervention | Passive | Falls; Fallers |
| Ling 2021 [104] | 31 | Community & RAC |  | Vitamin D |  | Falls |
| Liu et al, 2017 [57] | 22 | Community | Reduced physical ability | Exercise | Passive | Falls |
| Lomas-Vega 2017 [58] | 10 | Community & RAC |  | Exercise | Active | Falls |
| Mackenzie 2020 [59] | 19 | Community |  | Multifactorial intervention | Passive | Fallers |
| Maltais 2019 [60] | 40 | Community & RAC |  | Exercise | Active | Falls; Fallers; Fall-related fractures |
| Mañas 2021 [61] | 21 | Community |  | Exercise | Active | Falls; Fallers |
| Mansfield 2015 [62] | 8 | Community |  | Exercise | Active | Falls; Fallers |
| Manti 2009 [63] | 10 | Community |  | Exercise | Passive | Falls |
| Mat 2015 [64] | 15 | Community & RAC | Knee osteoarthritis | Exercise; Multifactorial intervention | Unclear | Falls |
| Mattle 2020 [65] | 29 | Community |  | Exercise | Active | Falls |
| Mayo-Wilson 2014 [66] | 64 | Community | Dementia | Multifactorial intervention | Passive | Falls; Fallers |
| Michael 2010 [67] | 54 | Community |  | Multifactorial intervention; Exercise; Vitamin D; Other; Medication review; Environmental; Education | Passive | Falls |
| Morello 2019 [69] | 12 | Community |  | Multifactorial intervention | Unclear | Falls; Fallers; Fall-related fractures |
| Moreno-Segura 2018 [70] | 15 | Community & RAC |  | Exercise | Passive | Falls |
| Murad 2011 [71] | 26 | Community & RAC |  | Vitamin D | Passive | Falls |
| Nørgaard 2021 [72] | 11 | Community |  | Exercise | Unclear | Falls; Fallers; Fall-related fractures |
| Okubo 2017 [73] | 16 | Community & RAC |  | Exercise | Unclear | Falls; Fallers |
| Schoberer 2020 [74] | 18 | RAC |  | Exercise | Passive | Falls; Fallers |
| Shekelle 2004 [75] | 40 | Community & RAC |  | Multifactorial intervention; Exercise; Environmental; Education | Unclear | Falls; Fallers |
| Sherrington 2020 [76] | 116 | Community |  | Exercise | Passive | Falls |
| Sibley 2021 [77] | 169 | Community & RAC |  | Exercise | Passive | Fallers |
| Silva 2013 [78] | 12 | RAC |  | Exercise | Passive | Falls |
| Solis-Navarro 2022 [105] | 26 | Community |  | Exercise | Unclear | Falls |
| Thanapluetiwong 2020 [79] | 53 | Community & RAC |  | Vitamin D | Unclear | Falls; Fall-related fractures |
| Thomas 2010 [80] | 7 | Community |  | Exercise | Passive | Falls |
| Thomas 2017 [81] | 283 | Community & RAC |  | Exercise; Mixed; Vitamin D; Multifactorial intervention | Passive | Fallers |
| Tricco 2019 [82] | 126 | Community & RAC |  | Education; Other | Passive | Fallers; Falls requiring hospitalisation |
| Tsang 2011 [83] | 13 | Community & RAC |  | Exercise | Passive; Active; Unclear | Falls |
| Veronese 2017 [84] | 6 | Community & RAC |  | Exercise | Unclear | Falls |
| Vlaeyen 2015 [68] | 13 | RAC |  | Multifactorial intervention; Quality improvement; Other | Unclear | Falls; Fallers |
| Wang 2021 [108] | 14 | RAC |  | Exercise | Passive | Falls; Fallers |
| Wang 2020 [86] | 20 | Community |  | Exercise | Passive | Fall-related fractures |
| Wang 2015 [87] | 30 | Community |  | Exercise | Passive | Falls |
| Weatherall 2004 [88] | 19 | Community |  | Exercise; Multifactorial intervention | Unclear | Fallers |
| Wiedenmann 2023 [106] | 66 | Community |  | Exercise | Unclear | Falls |
| Wong 2020 [89] | 12 | Community |  | Exercise | Unclear | Falls; Fall-related fractures |
| Wu 2017 [90] | 26 | Community |  | Vitamin D | Passive | Fallers |
| Wylie 2019 [91] | 7 | Community & RAC |  | Other; Multifactorial intervention | Passive | Falls |
| Xin 2018 [92] | 6 | Community | Osteoporosis | Exercise | Passive | Falls |
| Yu 2021 [93] | 11 | Community |  | Medication review | Passive | Fall-related fractures; Falls requiring hospitalisation |
| Zhang 2015 [94] | 7 | Community |  | Other; Multifactorial intervention | Passive | Falls |
| Zhao 2019 [95] | 25 | Community |  | Exercise | Passive | Fall-related fractures |
| Zhao 2017 [96] | 15 | Community |  | Exercise | Passive | Falls; Fall-related fractures |
| Zheng 2015 [97] | 9 | Community & RAC |  | Vitamin D | Passive | Falls |
| Ziebart 2020 [98] | 5 | Community |  | Multifactorial intervention | Passive | Falls |

*Specific population outside of age and setting

# References

1. Almutairi H, Stafford A, Etherton-Beer C, Flicker L: Optimisation of medications used in residential aged care facilities: a systematic review and meta-analysis of randomised controlled trials. *BMC Geriatr* 2020, 20(1):1-19.

2. Arnold CM, Sran MM, Harrison EL: Exercise for fall risk reduction in community-dwelling older adults: a systematic review. *Physiother Can* 2008, 60(4):358-372. <https://doi.org/10.3138/physio.60.4.358>.

3. Barker AL, Bird M-L, Talevski J: Effect of pilates exercise for improving balance in older adults: a systematic review with meta-analysis. *Arch Phys Med Rehabil* 2015, 96(4):715-723. <https://doi.org/10.1016/j.apmr.2014.11.021>.

4. Barreto PdS, Maltais M, Rosendahl E, Vellas B, Bourdel-Marchasson I, Lamb SE, Pitkala K, Roll Y, de Souto Barreto P: Exercise effects on falls, fractures, hospitalizations, and mortality in older adults with dementia: an individual-level patient data meta-analysis. *J Gerontol - Biol Sci Med Sci* 2021, 76(9):e203-e212. <https://doi.org/10.1093/gerona/glaa307>.

5. Beswick AD, Rees K, Ayis S, Gooberman-Hill R, Horwood J, Dieppe P, Ebrahim S: Complex interventions to improve physical function and maintain independent living in elderly people: a systematic review and meta-analysis. *The Lancet* 2008, 371(9614):725-735. <https://doi.org/10.1016/s0140-6736(08)60342-6>.

6. Bierma-Zeinstra SMA, Koes BW, Logghe IHJ, Verhagen AP, Rademaker ACHJ, van Rossum E, Faber MJ: The effects of Tai Chi on fall prevention, fear of falling and balance in older people: a meta-analysis. *Prev Med* 2010, 51(3):222-227.

7. Bischoff-Ferrari HA, Dawson-Hughes B, Staehelin HB, Orav JE, Stuck AE, Theiler R, Wong JB, Egli A, Kiel DP, Henschkowski J: Fall prevention with supplemental and active forms of Vitamin D: a meta-analysis of randomised controlled trials. *BMJ* 2009, 339:b3692. <https://doi.org/10.1136/bmj.b3692>.

8. Bischoff-Ferrari HA, Dawson-Hughes B, Willett WC, Staehelin HB, Bazemore MG, Zee RY, Wong JB, Bischoff-Ferrari HA, Dawson-Hughes B, Willett WC, Staehelin HB, Bazemore MG, Zee RY, Wong JB: Effect of Vitamin D on falls: a meta-analysis. *JAMA* 2004, 291(16):1999-2006. <https://doi.org/10.1001/jama.291.16.1999>.

9. Boll MJ, Grey A, Gamble GD, Reid IR: Vitamin D supplementation and falls: a trial sequential meta-analysis. *Lancet Diabetes Endocrinol* 2014, 2(7):573-580. <https://doi.org/10.1016/s2213-8587(14)70068-3>.

10. Bonner M, Capsey M, Batey J: A paramedic's role in reducing number of falls and fall-related emergency service use by over 65s: a systematic review. *British Paramedic Journal* 2021, 6(1):46-52. <https://doi.org/10.29045/14784726.2021.6.6.1.46>.

11. Cadore EL, Izquierdo M, Rodriguez-Manas L, Sinclair A: Effects of different exercise interventions on risk of falls, gait ability, and balance in physically frail older adults: a systematic review. *Rejuvenation Res* 2013, 16(2):105-114. <https://doi.org/10.1089/rej.2012.1397>.

12. Cameron ID, Dyer SM, Panagoda CE, Murray GR, Hill KD, Cumming RG, Kerse N: Interventions for preventing falls in older people in care facilities and hospitals. *Cochrane Database Syst Rev* 2018, 9(9):CD005465-CD005465. <https://doi.org/10.1002/14651858.cd005465.pub4>.

13. Campbell AJ, Robertson MC: Rethinking individual and community fall prevention strategies: a meta-regression comparing single and multifactorial interventions. *Age Ageing* 2007, 36(6):656-662. <https://doi.org/10.1093/ageing/afm122>.

14. Cao P-Y, Zhao Q-H, Xiao L, Xiao M-Z, Kong L-N: The effectiveness of exercise for fall prevention in nursing home residents: a systematic review meta-analysis. *J Adv Nurs* 2018, 74(11):2511-2522. <https://doi.org/10.1111/jan.13814>.

15. Caristia S, Campani D, Payedimarri AB, Faggiano F, Cannici C, Frontera E, Giarda G, Pisterzi S, Terranova L, Dal Molin A: Physical exercise and fall prevention: A systematic review and meta-analysis of experimental studies included in Cochrane reviews. *Geriatr Nurs* 2021, 42(6):1275-1286. <https://doi.org/10.1016/j.gerinurse.2021.06.001>.

16. Chamberlain-Carter J, Jackson J: Does resistance training reduce falls and improve quality of life in people with Parkinson's disease using strength training exercise programmes? *Phys Ther* 2021, 26(1):1-9. <https://doi.org/10.1080/10833196.2020.1814123>.

17. Chan JKY, Klainin-Yobas P, Chi Y, Gan JKE, Chow G, Wu XV: The effectiveness of e-interventions on fall, neuromuscular functions and quality of life in community-dwelling older adults: a systematic review and meta-analysis. *Int J Nurs Stud* 2021, 113:N.PAG-N.PAG. <https://doi.org/10.1016/j.ijnurstu.2020.103784>.

18. Chan WC, Fai Yeung JW, Man Wong CS, Wa Lam LC, Chung KF, Hay Luk JK, Wah Lee JS, Kin Law AC: Efficacy of physical exercise in preventing falls in older adults with cognitive impairment: a systematic review and meta-analysis. *J Am Med Dir Assoc* 2015, 16(2):149-154. <https://doi.org/10.1016/j.jamda.2014.08.007>.

19. Cheng P, Tan L, Ning P, Li L, Gao Y, Wu Y, Schwebel DC, Chu H, Yin H, Hu G: Comparative effectiveness of published interventions for elderly fall prevention: a systematic review and network meta-analysis. *Int J Environ Res Public Health* 2018, 15(3). <https://doi.org/10.3390%2Fijerph15030498>.

20. Choi M, Hector M: Effectiveness of intervention programs in preventing falls: a systematic review of recent 10 years and meta-analysis. *J Am Med Dir Assoc* 2012, 13(2):188.e113-121. <https://doi.org/10.1016/j.jamda.2011.04.022>.

21. Chua GT, Wong RY: Association between Vitamin D dosing regimen and fall prevention in long-term care seniors. *Can Geriatr J* 2011, 14(4):93-99. <https://doi.org/10.57700%2Fcgj.v14i4.23>.

22. Claudino JG, Afonso J, Sarvestan J, Lanza MB, Pennone J, Filho CAC, Serrão JC, Espregueira-Mendes J, Vasconcelos ALV, de Andrade MP, Rocha-Rodrigues S, Andrade R, Ramirez-Campillo R: Strength training to prevent falls in older adults: a systematic review with meta-analysis of randomized controlled trials. *J Clin Med* 2021, 10(14). <https://doi.org/10.3390/jcm10143184>.

23. Clemson L, Mackenzie L, Cumming RG, Ballinger C, Close JCT: Environmental interventions to prevent falls in community-dwelling older people: a meta-analysis of randomized trials. *J Aging Health* 2008, 20(8):954-971. <https://doi.org/10.1177/0898264308324672>.

24. Coussement J, De Paepe L, Schwendimann R, Denhaerynck K, Dejaeger E, Milisen K: Interventions for preventing falls in acute- and chronic-care hospitals: a systematic review and meta-analysis. *J Am Geriatr Soc* 2008, 56(1):29-36. <https://doi.org/10.1111/j.1532-5415.2007.01508.x>.

25. Dautzenberg L, Beglinger S, Tsokani S, Zevgiti S, Raijmann RCMA, Rodondi N, Scholten RJPM, Rutjes AWS, Di Nisio M, Emmelot‐Vonk M, Tricco AC, Straus SE, Thomas S, Bretagne L, Knol W, Mavridis D, Koek HL: Interventions for preventing falls and fall‐related fractures in community‐dwelling older adults: a systematic review and network meta‐analysis. *J Am Geriatr Soc* 2021, 69(10):2973-2984. <https://doi.org/10.1111/jgs.17375>.

26. De Kam D, Smulders E, Weerdesteyn V, Smits-Engelsman BCM: Exercise interventions to reduce fall-related fractures and their risk factors in individuals with low bone density: a systematic review of randomized controlled trials. *Osteoporosis Int* 2009, 20(12):2111-2125. <https://doi.org/10.1007/s00198-009-0938-6>.

27. Desapriya E, Subzwari S, Scime-Beltrano G, Samayawardhena LA, Pike I: Vision improvement and reduction in falls after expedited cataract surgery systematic review and metaanalysis. *J Cataract Refract Surg* 2010, 36(1):13-19. <https://doi.org/10.1016/j.jcrs.2009.07.032>.

28. Fernandez-Rodriguez R, Pozuelo-Carrascosa DP, Alvarez-Bueno C, Ferri-Morales A, Torres-Costoso A, Martinez-Vizcaino V: Pilates improves physical performance and decreases risk of falls in older adults: a systematic review and meta-analysis. *Physiotherapy* 2021, 112:163-177. <https://doi.org/10.1016/j.physio.2021.05.008>.

29. Fisseha B, Janakiraman B, Yitayeh A, Ravich, ran H: Effect of square stepping exercise for older adults to prevent fall and injury related to fall: systematic review and meta-analysis of current evidences. *J Exerc Rehabil* 2017, 13(1):23-29. <https://doi.org/10.12965/jer.1734924.462>.

30. Francis-Coad J, Etherton-Beer C, Burton E, Naseri C, Hill A-M: Effectiveness of complex falls prevention interventions in residential aged care settings: a systematic review. *JBI Database System Rev Implement Rep* 2018, 16(4):973-1002. <https://doi.org/10.11124/jbisrir-2017-003485>.

31. Garcia-Hermoso A, Ramirez-Velez R, Saez de Asteasu ML, Martinez-Velilla N, Zambom-Ferraresi F, Izquierdo M, Valenzuela PL, Lucia A, ro: Safety and effectiveness of long-term exercise interventions in older adults: a systematic review and meta-analysis of randomized controlled trials. *Sports Med* 2020, 50(6):1095-1106.

32. Garcia-Lopez H, Castro-Sanchez AM, Lara-Palomo IC, Obrero-Gaitan E, Nieto-Escamez FA, Cortes-Perez I: Non-immersive virtual reality to improve balance and reduce risk of falls in people diagnosed with parkinson's disease: a systematic review. *Brain Sci* 2021, 11(11):1435. <https://doi.org/10.3390/brainsci11111435>.

33. Gates S, Fisher JD, Cooke MW, Carter YH, Lamb SE: Multifactorial assessment and targeted intervention for preventing falls and injuries among older people in community and emergency care settings: systematic review and meta-analysis. *BMJ* 2008, 336(7636):130-133. <https://doi.org/10.1136/bmj.39412.525243.BE>.

34. Gomersall SR, Hatton A, Brauer S, G. r, Teng B: Combined group and home exercise programmes in community-dwelling falls-risk older adults: systematic review and meta-analysis. *Physiother Res Int* 2020, 25(3):e1839. <https://doi.org/10.1002/pri.1839>.

35. Goodwin VA, Abbott RA, Whear R, Bethel A, Ukoumunne OC, Thompson-Coon J, Stein K: Multiple component interventions for preventing falls and fall-related injuries among older people: systematic review and meta-analysis. *BMC Geriatr* 2014, 14(1):15-15. <https://doi.org/10.1186/1471-2318-14-15>.

36. Guirguis-Blake JM, Michael YL, Perdue LA, Coppola EL, Beil TL: Interventions to prevent falls in older adults: updated evidence report and systematic review for the US preventive services task force. *JAMA* 2018, 319(16):1705-1716. <https://doi.org/10.1001/jama.2017.21962>.

37. Gulka HJ, Patel V, Arora T, McArthur C, Iaboni A: Efficacy and generalizability of falls prevention interventions in nursing homes: a systematic review and meta-analysis. *J Am Med Dir Assoc* 2020, 21(8):1024-1024. <https://doi.org/10.1016/j.jamda.2019.11.012>.

38. Guo J-L, Tsai Y-Y, Liao J-Y, Tu H-M, Huang C-M: Interventions to reduce the number of falls among older adults with/without cognitive impairment: an exploratory meta-analysis. *Int J Geriatr Psychiatry* 2014, 29(7):661-669. <https://doi.org/10.1002/gps.4056>.

39. Gutiérrez-Robledo LM, Villasís-Keever MA, Avila-Avila A, Medina-Campos RH, Castrejón-Pérez RC, García-Peña C: Effect of cataract surgery on frequency of falls among older persons: a systematic review and meta-analysis. *J Ophthalmol* 2021:1-7. <https://doi.org/10.1155%2F2021%2F2169571>.

40. Haines TP, Lee D-CA, Etherton-Beer C, McPhail S, Morris ME, Flicker L, Netto J, Francis-Coad J, Shorr R, Naseri C, Hill A-M: Reducing falls in older adults recently discharged from hospital: a systematic review and meta-analysis. *Age Ageing* 2018, 47(4):512-519. <https://doi.org/10.1093/ageing/afy043>.

41. Harling A, Simpson JP: A systematic review to determine the effectiveness of Tai Chi in reducing falls and fear of falling in older adults. *Phys Ther Rev* 2008, 13(4):237-248. <https://doi.org/10.1179/174328808X309241>.

42. Herbert RD, Cumming RG, Lord SR, Whitney JC, Close JCT, Sherrington C: Effective exercise for the prevention of falls: a systematic review and meta-analysis. *J Am Geriatr Soc* 2008, 56(12):2234-2243. <https://doi.org/10.1111/j.1532-5415.2008.02014.x>.

43. Hill KD, Farrier K, Burton E, Suttanon P, Lin S-I, Tsang WWN, Ashari A, Hamid TAA: What works in falls prevention in Asia: a systematic review and meta-analysis of randomized controlled trials. *BMC Geriatr* 2018, 18(1):3. <https://doi.org/10.1186/s12877-017-0683-1>.

44. Hill KD, Hunter SW, Batchelor FA, Cavalheri V, Burton E: Individualized home-based exercise programs for older people to reduce falls and improve physical performance: a systematic review and meta-analysis. *Maturitas* 2015, 82(1):72-84. <https://doi.org/10.1016/j.maturitas.2015.04.005>.

45. Hopewell S, Adedire O, Copsey BJ, Boniface GJ, Sherrington C, Clemson L, Close JC, Lamb SE: Multifactorial and multiple component interventions for preventing falls in older people living in the community. *The Cochrane database of systematic reviews* 2018, 7:CD012221.

46. Hopewell S, Copsey B, Nicolson P, Adedire B, Boniface G, Lamb S: Multifactorial interventions for preventing falls in older people living in the community: a systematic review and meta-analysis of 41 trials and almost 20 000 participants. *Br J Sports Med* 2019. <https://doi.org/10.1136/bjsports-2019-100732>.

47. Huang Z-G, Feng Y-H, Li Y-H, Lv C-S: Systematic review and meta-analysis: Tai Chi for preventing falls in older adults. *BMJ Open* 2017, 7(2):e013661. <https://doi.org/10.1136/bmjopen-2016-013661>.

48. Jepsen DB, Thomsen K, Hansen S, Jørgensen NR, Masud T, Ryg J: Effect of whole-body vibration exercise in preventing falls and fractures: a systematic review and meta-analysis. *BMJ Open* 2017, 7(12). <https://doi.org/10.1136/bmjopen-2017-018342>.

49. Kalyani RR, Stein B, Valiyil R, Manno R, Maynard JW, Crews DC: Vitamin D treatment for the prevention of falls in older adults: systematic review and meta-analysis. *J Am Geriatr Soc* 2010, 58(7):1299-1310. <https://doi.org/10.1111%2Fj.1532-5415.2010.02949.x>.

50. Keay L, Dillon L, Sherrington C, Clemson L, Ramulu P: A systematic review and meta-analysis of exercise-based falls prevention strategies in adults aged 50+ years with visual impairment. *Ophthalmic Physiol Opt* 2018, 38(4):456-467. <https://doi.org/10.1111/opo.12562>.

51. Kua C-H, Mak VSL, Huey Lee SW: Health outcomes of deprescribing interventions among older residents in nursing homes: a systematic review and meta-analysis. *J Am Med Dir Assoc* 2019, 20(3):362-362. <https://doi.org/10.1016/j.jamda.2018.10.026>.

52. Latham NK, Anderson CS, Reid IR: Effects of Vitamin D supplementation on strength, physical performance, and falls in older persons: a systematic review. *J Am Geriatr Soc* 2003, 51(9):1219-1226. <https://doi.org/10.1046/j.1532-5415.2003.51405.x>.

53. Lee J, Negm A, Peters R, Wong EKC, Holbrook A: Deprescribing fall-risk increasing drugs (FRIDs) for the prevention of falls and fall-related complications: a systematic review and meta-analysis. *BMJ Open* 2021, 11(2). <https://doi.org/10.1136/bmjopen-2019-035978>.

54. Lee SH, Kim HS: Exercise Interventions for preventing falls among older people in care facilities: a meta-analysis. *Worldviews Evid Based Nurs* 2017, 14(1):74-80. <https://doi.org/10.1111/wvn.12193>.

55. Lee SH, Yu S: Effectiveness of multifactorial interventions in preventing falls among older adults in the community: a systematic review and meta-analysis. *Int J Nurs Stud* 2020, 106:N.PAG-N.PAG. <https://doi.org/10.1016/j.ijnurstu.2020.103564>.

56. Li F, Harmer P, Eckstrom E, Ainsworth BE, Fitzgerald K, Voit J, Chou L-S, Welker FL, Needham S: Efficacy of exercise-based interventions in preventing falls among community-dwelling older persons with cognitive impairment: is there enough evidence? An updated systematic review and meta-analysis. *Age Ageing* 2021, 50(5):1557-1568. <https://doi.org/10.1093/ageing/afab110>.

57. Liu C-J, Chang W-P, Araujo de Carvalho I, Savage KEL, Radford LW, Amuthavalli Thiyagarajan J: Effects of physical exercise in older adults with reduced physical capacity: meta-analysis of resistance exercise and multimodal exercise. *Int J Rehabil Res* 2017, 40(4):303-314. <https://doi.org/10.1097/mrr.0000000000000249>.

58. Lomas-Vega R, Obrero-Gaitán E, Molina-Ortega FJ, Del-Pino-Casado R: Tai Chi for risk of falls: a meta-analysis. *J Am Geriatr Soc* 2017, 65(9):2037-2043. <https://doi.org/10.1111/jgs.15008>.

59. Mackenzie L, Beavis AM, Tan ACW, Clemson L: Systematic review and meta-analysis of intervention studies with general practitioner involvement focused on falls prevention for community-dwelling older people. *J Aging Health* 2020. <https://doi.org/10.1177/0898264320945168>.

60. Maltais M, De Souto Barreto P, Roll Y, Vellas B: Association of long-term exercise training with risk of falls, fractures, hospitalizations, and mortality in older adults: a systematic review and meta-analysis. *JAMA Intern Med* 2019, 179(3):394-405. <https://doi.org/10.1001/jamainternmed.2018.5406>.

61. Mañas A, Gómez-Redondo P, Valenzuela PL, Morales JS, Lucía A, Ara I: Unsupervised home-based resistance training for community-dwelling older adults: a systematic review and meta-analysis of randomized controlled trials. *Ageing Res Rev* 2021, 69. <https://doi.org/10.1016/j.arr.2021.101368>.

62. Mansfield A, Wong JS, Bryce J, Knorr S, Patterson KK: Does perturbation-based balance training prevent falls? Systematic review and meta-analysis of preliminary randomized controlled trials. *Phys Ther* 2015, 95(5):700-709. <https://doi.org/10.2522/ptj.20140090>.

63. Manti EG, Ntinapogias AG, Negri E, Szczerbinska K, Petridou ET: What works better for community-dwelling older people at risk to fall? A meta-analysis of multifactorial versus physical exercise-alone interventions. *J Aging Health* 2009, 21(5):713-729. <https://doi.org/10.1177/0898264309338298>.

64. Mat S, Tan MPi, Kamaruzzaman SBa, Ng CTe: Physical therapies for improving balance and reducing falls risk in osteoarthritis of the knee: a systematic review. *Age Ageing* 2015, 44(1):16-24. <https://doi.org/10.1093/ageing/afu112>.

65. Mattle M, Chocano-Bedoya PO, Fischbacher M, Meyer U, Abderhalden LA, Lang W, Mansky R, Bischoff-Ferrari HA, Kressig RW, Steurer J, Orav EJ: Association of dance-based mind-motor activities with falls and physical function among healthy older adults: a systematic review and meta-analysis. *JAMA Netw Open* 2020, 3(9):17688. <https://doi.org/10.1001/jamanetworkopen.2020.17688>.

66. Mayo-Wilson E, Grant S, Burton J, Parsons A, Underhill K, Montgomery P: Preventive home visits for mortality, morbidity, and institutionalization in older adults: a systematic review and meta-analysis. *PLoS ONE* 2014, 9(3). <https://doi.org/10.1371/journal.pone.0089257>.

67. Michael YL, Whitlock EP, Lin JS, O'Connor EA, Gold R, Fu R: Primary care-relevant interventions to prevent falling in older adults: a systematic evidence review for the U.S. Preventive Services Task Force. *Ann Intern Med* 2010, 153(12):815-825. <https://doi.org/10.7326/0003-4819-153-12-201012210-00008>.

68. Vlaeyen E, Coussement J, Leysens G, Van der Elst E, Delbaere K, Cambier D, Denhaerynck K, Goemaere S, Wertelaers A, Dobbels F: Characteristics and effectiveness of fall prevention programs in nursing homes: a systematic review and meta‐analysis of randomized controlled trials. *J Am Geriatr Soc* 2015, 63(2):211-221. <https://doi.org/10.1111/jgs.13254>.

69. Morello RT, Behm K, Egan A, Ayton D, Barker AL, Soh S-E, Hill K, Flicker L, Etherton-Beer CD, Arendts G, Waldron N, Redfern J, Haines T, Lowthian J, Nyman SR, Cameron P, Fairhall N: Multifactorial falls prevention programmes for older adults presenting to the emergency department with a fall: systematic review and meta-analysis. *Inj Prev* 2019, 25(6):557-564. <https://doi.org/10.1136/injuryprev-2019-043214>.

70. Moreno-Segura N, Igual-Camacho C, Ballester-Gil Y, Blasco-Igual MC, Blasco JM: The effects of the pilates training method on balance and falls of older adults: a systematic review and meta-analysis of randomized controlled trials. *J Aging Phys Act* 2018, 26(2):327-344. <https://doi.org/10.1123/japa.2017-0078>.

71. Murad MH, Elamin KB, Abu Elnour NO, Elamin MB, Alkatib AA, Fatourechi MM, Almandoz JP, Mullan RJ, Lane MA, Liu H, Erwin PJ, Hensrud DD, Montori VM: Clinical review: the effect of vitamin D on falls: a systematic review and meta-analysis. *J Clin Endocrinol Metab* 2011, 96(10):2997-3006. <https://doi.org/10.1210/jc.2011-1193>.

72. Nørgaard JE, Jorgensen MG, Ryg J, Andreasen J, Danielsen MB, Steiner DK, Andersen S: Effects of gait adaptability training on falls and fall-related fractures in older adults: a systematic review and meta-analysis. *Age Ageing* 2021, 50(6):1914-1924. <https://doi.org/10.1093/ageing/afab105>.

73. Okubo Y, Schoene D, Lord SR: Step training improves reaction time, gait and balance and reduces falls in older people: a systematic review and meta-analysis. *Br J Sports Med* 2017, 51(7):586-593. <https://doi.org/10.1136/bjsports-2015-095452>.

74. Schoberer D, Breimaier HE: Meta‐analysis and GRADE profiles of exercise interventions for falls prevention in long‐term care facilities. *J Adv Nurs* 2020, 76(1):121-134. <https://doi.org/10.1111%2Fjan.14238>.

75. Shekelle PG, Morton SC, Mojica WA, Maglione M, Suttorp MJ, Roth EA, Rubenstein LZ, Chang JT: Interventions for the prevention of falls in older adults: systematic review and meta-analysis of randomised clinical trials. *BMJ* 2004, 328(7441):680-683. <https://doi.org/10.1136/bmj.328.7441.680>.

76. Sherrington C, Fairhall N, Kwok W, Wallbank G, Tiedemann A, Michaleff ZA, Ng CACM, Bauman A: Evidence on physical activity and falls prevention for people aged 65+ years: systematic review to inform the WHO guidelines on physical activity and sedentary behaviour. *Int J Behav Nutr Phys Act* 2020, 17(1):N.PAG-N.PAG. <https://doi.org/10.1186/s12966-020-01041-3>.

77. Sibley KM, Thomas SM, Veroniki AA, Rodrigues M, Hamid JS, Lachance CC, Cogo E, Khan PA, Riva JJ, Thavorn K, MacDonald H, Holroyd-Leduc J, Feldman F, Kerr GD, Jaglal SB, Straus SE, Tricco AC: Comparative effectiveness of exercise interventions for preventing falls in older adults: a secondary analysis of a systematic review with network meta-analysis. *Exp Gerontol* 2021, 143. <https://doi.org/10.1016/j.exger.2020.111151>.

78. Silva RB, Eslick GD, Duque G: Exercise for falls and fracture prevention in long term care facilities: a systematic review and meta-analysis. *J Am Med Dir Assoc* 2013, 14(9):685-689.e682. <https://doi.org/10.1016/j.jamda.2013.05.015>.

79. Thanapluetiwong S, Chewcharat A, Takkavatakarn K, Praditpornsilpa K, Eiam-Ong S, Susantitaphong P: Vitamin D supplement on prevention of fall and fracture: a meta-analysis of randomized controlled trials. *Medicine* 2020, 99(34):e21506. <https://doi.org/10.1097/md.0000000000021506>.

80. Thomas S, Mackintosh S, Halbert J: Does the ‘Otago exercise programme’ reduce mortality and falls in older adults? A systematic review and meta-analysis. *Age Ageing* 2010, 39(6):681-687. <https://doi.org/10.1093/ageing/afq102>.

81. Thomas SM, Veroniki AA, Hamid JS, Cogo E, Khan PA, Robson R, MacDonald H, Wilson C, Kerr GD, Hui W, Tricco AC, Strifler L, Sibley KM, Riva JJ, Thavorn K, Holroyd-Leduc J, Feldman F, Majumdar SR, Jaglal SB, Straus SE: Comparisons of interventions for preventing falls in older adults: a systematic review and meta-analysis. *JAMA* 2017, 318(17):1687-1699. <https://doi.org/10.1001/jama.2017.15006>.

82. Tricco AC, Thomas SM, Veroniki AA, Hamid JS, Cogo E, Strifler L, Khan PA, Sibley KM, Robson R, MacDonald H, Riva JJ, Thavorn K, Wilson C, Holroyd-Leduc J, Kerr GD, Feldman F, Majumdar SR, Jaglal SB, Hui W, Straus SE: Quality improvement strategies to prevent falls in older adults: a systematic review and network meta-analysis. *Age Ageing* 2019, 48(3):337-346. <https://doi.org/10.1093/ageing/afy219>.

83. Tsang HWH, Leung DPK, Chan CKL, William WNT, Jones AYM: Tai chi as an intervention to improve balance and reduce falls in older adults: a systematic and meta-analytical review. *Altern Ther Health Med* 2011, 17(1):40-48.

84. Veronese N, Maggi S, Schofield P, Stubbs B: Dance movement therapy and falls prevention. *Maturitas* 2017, 102:1-5. <https://doi.org/10.1016/j.maturitas.2017.05.004>.

85. Wang F, Tian B: The effectiveness of physical exercise type and length to prevent falls in nursing homes: a systematic review and meta‐analysis. *J Clin Nurs* 2022, 31(1-2):32-42. <https://doi.org/10.1111/jocn.15942>.

86. Wang Q, Shen Y, Yao P, Chen J, Zhou Y, Gu Y, Qian Z, Cao X, Jiang X: Effectiveness of exercise intervention on fall-related fractures in older adults: a systematic review and meta-analysis of randomized controlled trials. *BMC Geriatri* 2020, 20(1):322. <https://doi.org/10.1186/s12877-020-01721-6>.

87. Wang X, Pi Y, Chen P, Liu Y, Wang R, Chan C: Cognitive motor interference for preventing falls in older adults: a systematic review and meta-analysis of randomised controlled trials. *Age Ageing* 2015, 44(2):205-212. <https://doi.org/10.1093/ageing/afu175>.

88. Weatherall M: Prevention of falls and fall-related fractures in community-dwelling older adults: a meta-analysis of estimates of effectiveness based on recent guidelines. *Intern Med J* 2004, 34(3):102-108. <https://doi.org/10.1111/j.1444-0903.2004.t01-15-.x>.

89. Wong RMY, Chong KC, Law SW, Ho WT, Li J, Chui CS, Chow SKH, Cheung WH: The effectiveness of exercises on fall and fracture prevention amongst community elderlies: a systematic review and meta-analysis. *J Orthop* 2020, 24:58-65. <https://doi.org/10.1016/j.jot.2020.05.007>.

90. Wu H, Pang Q: The effect of Vitamin D and calcium supplementation on falls in older adults: a systematic review and meta-analysis. *Orthopade* 2017, 46(9):729-736. <https://doi.org/10.1007/s00132-017-3446-y>.

91. Wylie G, Torrens C, Campbell P, Frost H, Gordon AL, Menz HB, Skelton DA, Sullivan F, Witham MD, Morris J: Podiatry interventions to prevent falls in older people: a systematic review and meta-analysis. *Age Ageing* 2019, 48(3):327-336. <https://doi.org/10.1093/ageing/afy189>.

92. Xin Z, Hui D, Xiaoping S, Qingliang LEI: Effect of balance training on falls in patients with osteoporosis: a systematic review and meta-analysis. *J Rehabil Med* 2018, 50(7):577-581. <https://doi.org/10.2340/16501977-2334>.

93. Yu M, Zecevic A, A. r, Hunter SW, Wenxin M, Tirona RG: Medication review in preventing older adults' fall-related injury: a systematic review and meta-analysis. *Can Geriatr J* 2021, 24(3):237-250. <https://doi.org/10.5770/cgj.24.478>.

94. Zhang XY, Shuai J, Li LP: Vision and relevant risk factor interventions for preventing falls among older people: a network meta-analysis. *Sci Rep* 2015, 5. <https://doi.org/10.1038/srep10559>.

95. Zhao R, Bu W, Chen X: The efficacy and safety of exercise for prevention of fall-related injuries in older people with different health conditions, and differing intervention protocols: a meta-analysis of randomized controlled trials. *BMC Geriatr* 2019, 19(1):1-12. <https://doi.org/10.1186/s12877-019-1359-9>.

96. Zhao R, Feng F, Wang X: Exercise interventions and prevention of fall-related fractures in older people: a meta-analysis of randomized controlled trials. *Int J Epidemiol* 2017, 46(1):149-161. <https://doi.org/10.1093/ije/dyw142>.

97. Zheng YT, Cui QQ, Hong YM, Yao WG: A meta-analysis of high dose, intermittent vitamin D supplementation among older adults. *PLoS ONE* 2015, 10(1). <https://doi.org/10.1371/journal.pone.0115850>.

98. Ziebart C, Bobos P, Furtado R, MacDermid JC, Bryant D, Szekeres M, Suh N: The efficacy of fall hazards identification on fall outcomes: a systematic review with meta-analysis. *Arch Rehabil Res Clin Transl* 2020, 2(3):100065. <https://doi.org/10.1016%2Fj.arrct.2020.100065>.

99. Cao Y-t, Wang J-j, Yang Y-t, Zhu S-j, Zheng L-d, Lu W-w, Zhu R, Wu T: Effect of home-based exercise programs with e-devices on falls among community-dwelling older adults: a meta-analysis. *J Comp Eff Res* 2022, 11(16):1201-1217. <https://doi.org/10.2217/cer-2022-0130>.

100. Clemson L, Stark S, Pighills AC, Fairhall NJ, Lamb SE, Ali J, Sherrington C: Environmental interventions for preventing falls in older people living in the community. *Cochrane Database Syst Rev* 2023(3). <https://doi.org/10.1002/14651858.CD013258.pub2>.

101. Devasahayam AJ, Farwell K, Lim B, Morton A, Fleming N, Jagroop D, Aryan R, Saumur TM, Mansfield A: The effect of reactive balance training on falls in daily life: an updated systematic review and meta-analysis. *Phys Ther* 2023, 103(1):pzac154. <https://doi.org/10.1093/ptj/pzac154>.

102. Jehu DA, Davis JC, Gill J, Oke O, Liu-Ambrose T: The effect of exercise on falls in people living with dementia: a systematic review. *J Alzheimers Dis* 2023(Preprint):1-19. <https://doi.org/10.3233/jad-221038>.

103. Kong SH, Jang HN, Kim JH, Kim SW, Shin CS: Effect of Vitamin D supplementation on risk of fractures and falls according to dosage and interval: a meta-analysis. *Endocrinol Metab (Seoul)* 2022, 37(2):344-358. <https://doi.org/10.3803/enm.2021.1374>.

104. Ling Y, Xu F, Xia X, Dai D, Xiong A, Sun R, Qiu L, Xie Z: Vitamin D supplementation reduces the risk of fall in the vitamin D deficient elderly: an updated meta-analysis. *Clin Nutr* 2021, 40(11):5531-5537. <https://doi.org/10.1016/j.clnu.2021.09.031>.

105. Solis-Navarro L, Gismero A, Fernández-Jané C, Torres-Castro R, Solá-Madurell M, Bergé C, Pérez LM, Ars J, Martín-Borràs C, Vilaró J: Effectiveness of home-based exercise delivered by digital health in older adults: a systematic review and meta-analysis. *Age Ageing* 2022, 51(11):afac243. <https://doi.org/10.1093/ageing/afac243>.

106. Wiedenmann T, Held S, Rappelt L, Grauduszus M, Spickermann S, Donath L: Exercise based reduction of falls in communitydwelling older adults: a network meta-analysis. *Eur Rev Aging Phys Act* 2023, 20(1):1. <https://doi.org/10.1186/s11556-023-00311-w>.

107. Huang SF, Yang TT, Chen SF, Lu LT, Liao JY: Effects of fall prevention programs for older adults on fall-related injuries: a systematic review and meta-analysis. *J Res Educ Sci* 2018, 63(2):163-186. <http://dx.doi.org/10.6209/JORIES.201806_63(2).0007>.

108. Wang F, Tian B: The effectiveness of physical exercise type and length to prevent falls in nursing homes: a systematic review and meta-analysis. *J Clin Nurs* 2021. <https://doi.org/10.1111/jocn.15942>.
